# Extended DEMATEL method with intuitionistic fuzzy information: A case of electric vehicles

**DOI:** 10.1371/journal.pone.0314650

**Published:** 2024-12-19

**Authors:** Qiwen Ye

**Affiliations:** School of Economics & Management, South China Normal University, Guangzhou, China; Gonbad Kavous University, ISLAMIC REPUBLIC OF IRAN

## Abstract

The Decision-Making Trial and Laboratory (DEMATEL) methodology excels in the analysis of interdependent factors within complex systems, with correlation data typically presented in crisp values. Nevertheless, the judgments made by decision-makers often possess a degree of fuzziness and uncertainty, rendering the sole reliance on precise values inadequate for representing real-world scenarios. To address this issue, our study extends the DEMATEL approach to more effectively and efficiently handle intuitionistic fuzzy information, which denotes the factor correlation information from decision-makers in the form of intuitionistic fuzzy terms. The paper aggregates the intuitionistic fuzzy correlation information from each decision-maker, employing operators designed for managing intuitionistic fuzzy numbers. The significance and categorization of factors are determined through intuitionistic fuzzy matrix operations. Additionally, a causal and effect diagram is constructed to elucidate the distinct roles of these factors. Finally, this study illustrates the applicability of our proposed method with a real-world case in the context of electric vehicles (EVs). The study’s results identify four cause factors and six effect factors within EV battery technology. The identification and categorization of these factors will assist EV companies in implementing targeted measures to foster the advancement of the battery technology.

## 1 Introduction

The engineering [1, 2], economic and social systems [3, 4] are all complex and dynamic systems. Systematic elements interact and profoundly change the world via casual relationships, such as the effect of greenhouse gases on global climate; the dynamic process of resource allocation occurring in the transportation system [5]. Some of these casual relationships among elements are conceivably straight forward whereas the others are complicated to identify and may even impose perils on our survival. Facing such situations, decision-makers are growingly apprehensive that the traditional tools not only prove ineffective in addressing their enduring challenges but might, in fact, exacerbate them. Regrettably, earnest attempts to resolve pressing issues frequently give rise to unforeseen and adverse consequences. Therefore, the analysis of causal relationships among systematic elements raises an urgent challenge.

The Decision-Making Trial and Evaluation Laboratory (DEMATEL) technique, originally developed by the Battelle Memorial Institute in collaboration with the Geneva Research Centre [6], is designed to elucidate intricate interconnections within dynamic systems [7]. DEMATEL leverages graph theory and matrix tools, representing a widely employed approach for comprehensive element assessments. By scrutinizing the logical interdependencies and direct impacts among system elements, DEMATEL effectively discerns the significance of these elements and classifies them as either effect factors or cause factors [8]. The significance of an element pertains to its role and the degree of control it necessitates. This categorization facilitates the analysis of intricate relationships between system elements. Essentially, DEMATEL excels in examining bidirectional and transitive causal associations among numerous system elements in complex, dynamic systems, employing causal and effect diagrams to visualize system causality.

In recent decades, the DEMATEL method becomes prevailing in different areas such as operation management [9], innovation policy portfolio reconfiguration [10], human resource management [11], knowledge management [12], transportation management [13], and environmental economics decision [14, 15]. In addition to researching DEMATEL applications, scholars are continuously improving and expanding the method to increase its accuracy and applicability. This line of research can be categorized into two main streams. One stream involves combining DEMATEL with other decision-making methods, primarily to better adapt to specific decision-making scenarios and stratify different decision-making requirements. For example, Ortiz-Barrios et al. (2020), Karasan et al. (2022), WH El-Garaihy (2021) integrated the analytic hierarchy process (AHP) or data envelopment analysis (DEA) with DEMATEL [16–18]. The factor hierarchy analysis of AHP and DEA can be complemented with DEMATEL’s factor association analysis to comprehensively analyze the logical associations of decision-making factors. Similarly, research such as Chen (2021), Liang et al. (2022) combined interpretive structural modelling (ISM) with DEMATEL to enrich the analysis of factors’ relations [19, 20]. Furthermore, extending the DEMATEL method with the technique for order reference by similarity to ideal solution (TOPSIS) aids in optimizing sorting schemes [21, 22]. Scholars also extended DEMATEL with partial least squares (PLS) and Vise Kriterijumska Optimizacija Kompromisno Resenje (VIKOR), in order to reduce the difficulty of obtaining direct correlation matrices and solve decision-making problems with conflicting attributes [23–25].

The other research stream of extended DEMATEL focuses on the quantitative representation of expert preference information and is dominated by the gray DEMATEL and fuzzy DEMATEL. Grey DEMATEL combines gray system theory to extend the DEMATEL method for the incompleteness of decision-making information [14, 26]. Fuzzy DEMATEL, on the other hand, addresses the fuzziness and uncertainty of expert decision-making information, as decision makers prefer to apply linguistic terms to express their thoughts over crisp values due to fuzzy cognitions of human beings. In order to better handle the mentioned issue, different formats of fuzzy number are involved in previous scholarly efforts. Triangular fuzzy numbers, trapezoidal fuzzy numbers, Z-numbers, and 2-tuple linguistics are the most common fuzzy numbers integrated into DEMATEL, which can more scientifically portray the ambiguity and uncertainty of the experts in the DEMATEL judgment process and capture the fuzzy relationships between the factors. For example, Mahmoudi et al. (2019), Abdullah and Goh (2019), Çelik and Arslankaya (2023) tailored the fuzzy DEMATEL method with triangular fuzzy numbers for group decision making regarding the health care, waste management and quality management [27–29]. Compared with triangular fuzzy numbers, trapezoidal fuzzy numbers can be more flexible in dealing with complex fuzzy variables. Stephen and Felix (2023) applied DEMATEL in complex modifiable risk factors identification of cardiovascular disease with triangular fuzzy numbers [30]. Triangular fuzzy numbers are also employed in integrated multi-criteria decision-making methods, which involve complicated process [31, 32]. Similarly, Gaussian fuzzy numbers and Pythagorean fuzzy numbers are also commonly employed in extended DEMATEL methods [33–35]. The former accurately characterizes the distribution of fuzzy variables, thereby suitable for delineating continuous fuzzy variables. Conversely, the latter is capable of representing the fuzzy range of variables rather than fixed fuzzy values, thereby affording a degree of flexibility. However, it is unable to capture the distribution of variables within the fuzzy range.

Additionally, more complex fuzzy numbers have been integrated into the DEMATEL method, such as the commonly encountered 2-tuple linguistics, Z-numbers, and T-spherical fuzzy sets. In comparison to traditional single-value fuzzy numbers, 2-tuple linguistics, through the combination of membership degree and non-membership degree, offers a more precise depiction of the degree and scope of fuzziness. Suo et al. (2019) introduced a DEMATEL approach that incorporates 2-tuple linguistics, utilizing the 2-tuple linguistic representation model, serving the purpose of simultaneously determining the significance and categorization of infrastructures risk factors [36]. In addition, Zhang et al. (2020) used 2-tuple linguistic data to create an interval-value intuitionistic fuzzy sets and applied the DEMATEL method to analyze the dimensions and standards of youth unemployment risk factors [37]. Z-numbers can comprehensively consider the deterministic components and uncertainty range of numerical values, but the calculation process is relatively complex and depends heavily on parameter settings. Wang et al. (2021) extended the DEMATEL method with Z-numbers and proposed the Z-numbers power weighted average operator for evaluating the human error probability [38]. In the same vein, Z-numbers are also integrated into DEMATEL for handling fuzzy information in different decision-making scenarios, such as the value propositions evaluation of smart product-service systems and the indicator identification of hospital performance [39, 40]. The membership degree of T-spherical fuzzy sets gradually decreases as the distance between elements and the center of the fuzzy set increases, allowing it to better describe the fuzzy or uncertain relationship between elements and the fuzzy set. However, this characteristic also limits its ability to describe fuzziness, which may lead to poor performance in addressing certain complex fuzzy problems. Therefore, T-spherical fuzzy sets usually applied in complex integrated DEMATEL methods for handling partial fuzzy information in the decision problems. For example, this fuzzy set is common in the integrated multi-criteria decision-making methods, such as the TOPSIS-DEMATEL methods proposed by Eti et al. (2023), Özdemirci et al. (2023) [41, 42]. In addition, T-spherical fuzzy sets also employed in the DEMAEL methods combining with other fuzzy numbers, such as 2-tuple linguistic [43], Pythagorean fuzzy numbers [44].

The aforementioned fuzzy numbers are typically applied in engineering, decision analysis, control systems, and similar domains, primarily derived using mathematical methods or statistical analyses. They are suitable for problems that can be described through quantifiable data or models, rather than relying on subjective judgments from experts. However, in practical situations, decision-makers usually come from different areas, bringing varying levels of knowledge and expertise in their respective domains [45–48]. Consequently, decision-makers may not have enough expertise to precisely articulate their judgment regarding the objects or candidates, resulting in fuzziness and uncertainty in the process of group decision-making. Essentially, the mentioned fuzzy numbers are primarily defined by their membership degrees, making it challenging to quantify non-membership and hesitation degrees. This inherent limitation leads to initial information loss and subsequent decrease in precision. Under this circumstance, it is advisable to represent decision-maker evaluations through the application of intuitionistic fuzzy numbers [49, 50]. This approach is believed to effectively address the limitations mentioned above. Thus, using intuitionistic fuzzy numbers to convey the judgment of decision-makers is more suitable than relying on exact numerical values or conventional fuzzy variables [51]. Emerging literature has largely focused on the intuitionistic fuzzy sets (IFSs) theory and applied it to such areas as cluster analysis [52], medical diagnosis [53], decision making [54, 55], and pattern recognition [56–58].

Notwithstanding the pervasive use, there is a lack of an appropriate method to tackle the dependent factor analysis problem using intuitionistic fuzzy information. In several practical problems of dependent factor analysis such as risk factor analysis, adoption factor analysis and pricing factor analysis, decision-makers may need additional comprehension to express their judgment containing the information affirmation, negation and hesitation. Given this situation, it is conceivable to notice that the intuitionistic fuzzy information is more effective and appropriate for addressing the problems identified. However, the intuitionistic fuzzy information is not involved in the process of dependent factors analysis in existing literature. In addition, the prior fuzzy DEMATEL methods are incapable of dealing with the decision problems with the intuitionistic fuzzy information. In an effort to advance this line of research, this paper proposes a novel method to more efficiently and effectively cope with the pressing problem. To demonstrate the proposed method, this paper applies the extended DEMATEL method in factor identification of electric vehicle (EV). EV is the innovative vehicle for transportation sustainability and smart logistics, and its decision-making is under an intuitionistic fuzzy environment with uncertain technical factors and fuzzy judgement information.

The rest of this paper is structured as follows: Section 2 provides a concise review of the basic concepts and definitions of IFSs and the DEMATEL method. Subsequently, in Section 3, an expanded DEMATEL approach is introduced for analyzing the interrelationships among factors within the intuitionistic fuzzy context. The potential of the proposed method is illustrated through a real case in Section 4. Finally, Section 5 serves to summarize and highlight the characteristics and contribution of the proposed method.

## 2 Preliminaries

In this section, the author provides the illustration of the fundamental concepts and definitions related to the DEMATEL method and the IFSs.

### 2.1 The intuitionistic fuzzy set

The IFS proposed by Atanassov (1986) offers a viable approach to address vagueness. It is built on the foundation of classical fuzzy set theory [50].

**Definition 1** Let set *Y* is fixed. An IFS *A* in *Y* is defined as

$$A=\{<y,u_{A}(y),v_{A}(y)>|y\in Y\},$$ (1)

where *u*_*A*_(*y*),*v*_*A*_(*y*):*Y*→[0,1] are the membership function and non-membership function, respectively, with the condition 0≤*u*_*A*_(*y*)+*v*_*A*_(*y*)≤1.

For each IFS in *Y*, let’s call

$$\omega_{A}(y)={1-u}_{A}(y)-v_{A}(y),$$ (2)

the intuitionistic index *y* of in *A*. It represents the hesitancy degree of *y* to *A* [59].

The author observed that

$$0\leq \omega_{A}(y)\leq 1,\forall y\in Y.$$ (3)

For each fuzzy set *F* in *Y*, there is $\omega_{A}(y)={1-u}_{A}(y)-(1-u_{A}(y))=0,\forall y\in Y$. Hence, fuzzy sets are considered as the particular cases of IFSs.

For convenience, let’s denote an intuitionistic fuzzy number (IFN) [50, 60] by *a* = (*u*_*a*_,*v*_*a*_) where *u*_*a*_∈[0,1], *v*_*a*_∈[0,1], *u*_*a*_+*v*_*a*_≤1, and let *Θ* be the set of all IFNs.

The score of *S*(*a*) can be assessed using the score function *S* shown as [61]

$$S(a)=u_{a}+\frac{1+u_{a}-v_{a}}{2}\omega_{a},$$ (4)

where *S*(*a*) = [0,1].

For any three IFNs *a* = (*u*_*a*_,*v*_*a*_), $a_{1}=(u_{a_{1}},v_{a_{1}})$ and $a_{2}=(u_{a_{2}},v_{a_{2}})$ the following operational laws hold true.

(1) $a_{1}⨁a_{2}=(u_{a_{1}}+u_{a_{2}}-{u_{a_{1}}u}_{a_{2}},v_{a_{1}}v_{a_{2}})$,

(2) $a_{1}⨂a_{2}=(u_{a_{1}}u_{a_{2}},{v_{a_{1}}+v}_{a_{2}}-v_{a_{1}}v_{a_{2}})$,

(3) $\lambda a=(1-{\left(1-u_{a}\right)}^{\lambda },v_{a}^{\lambda })$,

(4) $a^{\lambda }=(u_{a}^{\lambda },1-{\left(1-v_{a}\right)}^{\lambda })$.

**Definition 2** Let $a_{j}=(u_{a_{j}},v_{a_{j}}),j=1,2,\ldots n$ be a collection of IFNs, and let the intuitionistic fuzzy weighted average (IFWA) [62], *IFWA*:*Θ*^*n*^→*Θ*, if

$$IFWA(a_{1},a_{2},\ldots ,a_{n})=(1-\prod_{j=1}^{n}{\left(1-u_{a_{j}}\right)}^{\delta_{j}},\prod_{j=1}^{n}{v_{a_{j}}}^{\delta_{j}}),$$ (5)

then *IFWA* is the intuitionistic fuzzy weighted averaging value, where *δ* = (*δ*_1_,*δ*_2_,…,*δ*_*n*_)^*T*^ is the weight vector of *a*_*j*_(*j* = 1,2,…,*n*) with *δ*_*j*_∈[0,1] and $\sum_{j=1}^{n}\delta_{j}=1$.

### 2.2 The DEMATEL method

This section will present the principal of the DEMATEL method. Suppose that the set of factors is *F* = {*F*_1_,*F*_2_,…,*F*_*n*_} the procedure steps of the method are as follow [63–65]:

***Steps 1*:** Establish the initial direct-relation matrix.

Let *z*_*ij*_ denotes the indicated degree of the dependence between factors *F*_*i*_ and *F*_*j*_. Particularly, there does not exist dependence between *F*_*i*_ and itself. Then the initial direct-relation matrix *Z* = [*z*_*ij*_]_*n*×*n*_ is constructed.

***Step 2*:** Calculate the normalized initial direct-relation matrix.

Normalize the initial direct-relation matrix *Z* with the following method and obtain the normalized initial direct-relation matrix *Y* = [*y*_*ij*_]_*n*×*n*_.

$$Y=Z/s\;and\;s=\underset{1\leq i\leq n}{\max}\left\{\sum_{j=1}^{j=n}z_{ij}\right\},i,j=1,2,\ldots ,n.$$ (6)

In (6), by eliminating all rows and columns related to the absorbing states, the sub-stochastic matrix *Y* is obtained from an absorbing Markov chain matrix, and equipped with the following two properties [66]:

(1) $\underset{\tau \to \infty }{\lim}Y^{\tau }=O$, where *O* is the null matrix;

(2) $\underset{\tau \to \infty }{\lim}{(Y^{1}+Y^{2}+\ldots +Y}^{\tau })=Y{\left(1-Y\right)}^{-1}$, where *I* is the identity matrix.

***Steps 3*:** Calculate the over-relation matrix.

Let *T* = [*t*_*ij*_]_*n*×*n*_ be over-relation matrix, and it can be obtained with

$$T=\underset{\tau \to \infty }{\lim}{(Y^{1}+Y^{2}+\cdots +Y}^{\tau })=Y{\left(1-Y\right)}^{-1},$$ (7)

where *t*_*ij*_ denotes the overall degree of correlation between factors *F*_*i*_ and *F*_*j*_.

***Steps 4*:** Determine the factor prominence and relations.

Let *c*_*i*_ represent the overall degree that factor *F*_*i*_ influences others and it can be expressed as

$$c_{i}=\sum_{j=1}^{n}t_{ij},i=1,2,\ldots ,n.$$ (8)

Let *h*_*i*_ denote the overall degree to which factor *F*_*i*_ is influenced by others. *h*_*i*_ can be obtained as follow

$$h_{i}=\sum_{j=1}^{n}t_{ji},i=1,2,\ldots ,n.$$ (9)

Let *D*_*i*_ be the prominence of factor *F*_*i*_ that determine the importance of factor *F*_*i*_ and it is

$$D_{i}=c_{i}+h_{i},i=1,2,\ldots ,n.$$ (10)

The larger the value of *D*_*i*_, the more significant factor *F*_*i*_ is.

Let *R*_*i*_ denote the relation of factor *F*_*i*_ an indicator for assessing the role of factor *F*_*i*_ and it is defined as

$$R_{i}=c_{i}-h_{i},i=1,2,\ldots ,n.$$ (11)

If *R*_*i*_>0, then *F*_*i*_ is a cause factor; if *R*_*i*_<0 then *F*_*i*_ is an effect factor.

***Steps 5*:** Build the causal and effect diagram.

A causal and effect diagram with the horizontal axis *D* and vertical axis *R* is constructed to visualize the importance and classification of all factors based on prominence *D*_*i*_ and relation *R*_*i*_.

## 3 The proposed method

**Fig 1:**
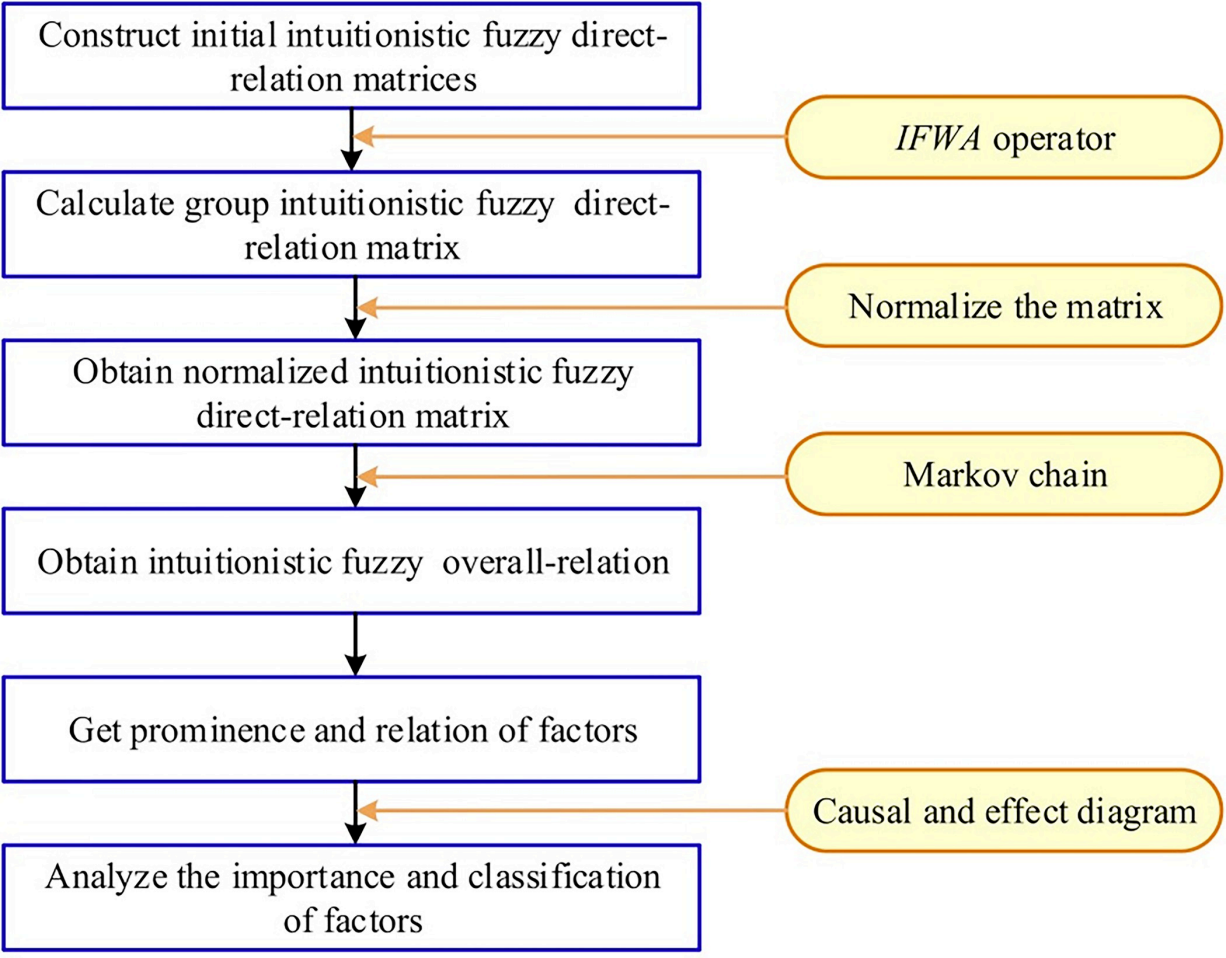
Resolution process of the presented problem.

In this section, an extended DEMATEL method with the IFSs is proposed to better identify the importance and classification of factors. A formal procedure of the proposed method is provided as illustrated in Fig 1. Firstly, the initial intuitionistic fuzzy direct-relation matrices are aggregated into a group intuitionistic fuzzy direct-relation matrix using *IFWA*. Then, normalized intuitionistic fuzzy direct-relation matrix and intuitionistic fuzzy over-relation matrix are constructed to derive the prominence and relation of factors. Furthermore, establish the importance of factors with respect to the prominences, and the classification of factors based on the relations. Finally, a causal and effect diagram is constructed based on the prominences and relations.

Let *E* = {*E*_1_,*E*_2_,…,*E*_*m*_} be a finite set of decision-makers and *E*_*k*_ (*k* = 1,2,…,*m*) represent the *k*^*th*^ decision-maker. The author makes the assumption that the decision makers share equal importance. *F* = {*F*_1_,*F*_2_,…,*F*_*n*_} is a finite set of factors and *F*_*i*_ (*i* = 1,2,…,*n*) denotes the *i*^*th*^ factor. Let $z_{ij}^{k}=(u_{ij}^{k},v_{ij}^{k})(k=1,2,\ldots ,m);i,j=1,2,\ldots ,n$ be the judgment of decision maker *E*_*k*_ on the correlation degree between factors *F*_*i*_ and *F*_*j*_. Particularly, $z_{ii}^{k}=(0,0)$ indicates that there is no correlation between *F*_*i*_ and itself. Then the initial intuitionistic fuzzy direct-relation matrix *Z*^*k*^ provided by decision-maker *E*_*k*_ can be built as follows.

$$Z^{k}=[z_{ij}^{k}]=[\begin{array}{c} F_{1}\\ F_{2}\\ \vdots \\ F_{n} \end{array}\begin{array}{cccc} F_{1} & F_{2} & \cdots & F_{n}\\ \left(0,0\right) & \left(u_{12}^{k},v_{12}^{k}\right) & \cdots & \left(u_{1n}^{k},v_{1n}^{k}\right)\\ \left(u_{21}^{k},v_{21}^{k}\right) & \left(0,0\right) & \cdots & \left(u_{2n}^{k},v_{2n}^{k}\right)\\ \vdots & \vdots & \ddots & \vdots \\ \left(u_{n1}^{k},v_{n1}^{k}\right) & \left(u_{n2}^{k},v_{n2}^{k}\right) & \cdots & \left(0,0\right) \end{array}]$$

k=1,2,…,m. (12)

Then, the author aggregates all the individual decision opinions into a group opinion, namely the group intuitionistic fuzzy decision matrix, which can be derived with Eq (5) and denoted as *Z*, i.e.,

$$Z=[\begin{array}{cccc} z_{11} & z_{12} & \cdots & z_{1n}\\ z_{21} & z_{22} & \cdots & z_{2n}\\ \vdots & \vdots & \ddots & \vdots \\ z_{n1} & z_{n2} & \cdots & z_{nn} \end{array}],$$ (13)

where $z_{ij}=({\bar{u}}_{ij},{\bar{v}}_{ij})=(1-\prod_{k=1}^{n}{\left(1-u_{ij}^{k}\right)}^{\frac{1}{n}},\prod_{k=1}^{n}{\left(v_{ij}^{k}\right)}^{\frac{1}{n}}),i,j=1,2,\ldots ,n$.

The normalized intuitionistic fuzzy direct-relation matrix of the group intuitionistic fuzzy direct-relation matrix *Z*, denoted as *Y* is given by

$$Y=[\begin{array}{cccc} y_{11} & y_{12} & \cdots & y_{1n}\\ y_{21} & y_{22} & \cdots & y_{2n}\\ \vdots & \vdots & \ddots & \vdots \\ y_{n1} & y_{n2} & \cdots & y_{nn} \end{array}],$$ (14)

where $y_{ij}=(u_{ij},v_{ij})=(\frac{1}{n}{\bar{u}}_{ij},\frac{1}{n}{\bar{v}}_{ij})$.

Two crisp values matrices from *Y* are obtained as follows:

$$U=\left[\begin{array}{ccc} u_{11} & \cdots & u_{1n}\\ \vdots & \ddots & \vdots \\ u_{n1} & \cdots & u_{nn} \end{array}\right],\;and\;V=\left[\begin{array}{ccc} v_{11} & \cdots & v_{1n}\\ \vdots & \ddots & \vdots \\ v_{n1} & \cdots & v_{nn} \end{array}\right].$$ (15)

The following proposition enables the computation of *Y* to be achieved by the multiplication of crisp matrices.

**Proposition 1** Let *Y*^τ^ = [(*y*_*ij*_)^τ^]_*n*×*n*_, where ${{(y}_{ij})}^{\tau }=({{(u}_{ij})}^{\tau },{{(v}_{ij})}^{\tau }),i,j=1,2,\ldots ,n$. Two matrices are further defined,

$$U^{\tau }={\left[{\left(u_{ij}\right)}^{\tau }\right]}_{n\times n}=[\begin{array}{cccc} {\left(u_{11}\right)}^{\tau } & {\left(u_{12}\right)}^{\tau } & \cdots & {\left(u_{1n}\right)}^{\tau }\\ {\left(u_{21}\right)}^{\tau } & {\left(u_{22}\right)}^{\tau } & \cdots & {\left(u_{2n}\right)}^{\tau }\\ \vdots & \vdots & \ddots & \vdots \\ {\left(u_{n1}\right)}^{\tau } & {\left(u_{n2}\right)}^{\tau } & \cdots & {\left(u_{nn}\right)}^{\tau } \end{array}],\;\mathrm{and}$$

$$V^{\tau }={\left[{\left(v_{ij}\right)}^{\tau }\right]}_{n\times n}=[\begin{array}{cccc} {\left(v_{11}\right)}^{\tau } & {\left(v_{12}\right)}^{\tau } & \cdots & {\left(v_{1n}\right)}^{\tau }\\ {\left(v_{21}\right)}^{\tau } & {\left(v_{22}\right)}^{\tau } & \cdots & {\left(v_{2n}\right)}^{\tau }\\ \vdots & \vdots & \ddots & \vdots \\ {\left(v_{n1}\right)}^{\tau } & {\left(v_{n2}\right)}^{\tau } & \cdots & {\left(v_{nn}\right)}^{\tau } \end{array}].$$ (16)

***Proof*** The proof is skipped due to its straightforwardness by using matrix multiplication.

**Proposition 2** $\underset{\tau \to \infty }{\lim}U^{\tau }=O,\;\underset{\tau \to \infty }{\lim}V^{\tau }=O,\;\underset{\tau \to \infty }{\lim}(I+U+U^{2}+\ldots +U^{\tau })={\left(I-U\right)}^{-1}$, and $\underset{\tau \to \infty }{\lim}(I+V+V^{2}+\cdots +V^{\tau })={\left(I-V\right)}^{-1}$.

***Proof*** The augmented matrix *U*′ as below is obtained by adding a row and a column to the matrix *U*. $U^{\prime }=[\begin{array}{cccc} & & & u_{1,n+1}\\ & U & & u_{2,n+1}\\ & & & \vdots \\ 0 & \cdots & 0 & 1 \end{array}]$, where *u*_1,*n*+1_,*u*_2,*n*+1_,…, *u*_*n*,*n*+1_ make *U*′ be a stochastic matrix. Since $\sum_{i=1}^{n}\sum_{j=1}^{n}u_{ij}<n$, there is at least one *u*_1,*n*+1_,*u*_2,*n*+1_,…, *u*_*n*,*n*+1_ is positive. Therefore, *U*′ is a stochastic matrix of an absorbing Markov chain and matrix *U* is the sub-stochastic matrix of *U*′. Let *ϱ*(*U*) be the spectral radius of matrix *U*, and the sufficient and necessary condition of *ϱ*(*U*)<1 is $\underset{\tau \to \infty }{\lim}U^{\tau }=O$ [65, 66]. Hence, there is $\underset{\tau \to \infty }{\lim}(I+U+U^{2}+\ldots +U^{\tau })={\left(I-U\right)}^{-1}$. $\underset{\tau \to \infty }{\lim}(I+V+V^{2}+\ldots +V^{\tau })={\left(I-V\right)}^{-1}$ can be proved with the analogous procedures.

The author defined the intuitionistic fuzzy over-relation matrix *T* as Eq (17), following the classical DEMATEL method [67]

$$T=\underset{\tau \to \infty }{\lim}\left(Y+Y^{2}+\cdots +Y^{\tau }\right).$$ (17)

**Proposition 3** Let *T* = [*t*_*ij*_]_*n*×*n*_,*i*,*j* = 1,2,…,*n*, where $t_{ij}=(t_{ij}^{1},t_{ij}^{2})$ then

$[t_{ij}^{1}{]}_{n\times n}=U{\left(I-U\right)}^{-1},i,j=1,2\ldots ,n$, and $[t_{ij}^{2}{]}_{n\times n}=V{\left(I-V\right)}^{-1},i,j=1,2\ldots ,n$.

***Proof*** By (17) and Propositions 1 and 2, there is

$${[t}_{ij}^{1}{]}_{n\times n}=\underset{\tau \to \infty }{\lim}(U+U^{2}+\cdots +U^{\tau })=\underset{\tau \to \infty }{\lim}U(I+U+U^{2}+\cdots +U^{\tau -1})=\underset{\tau \to \infty }{\lim}U(I+U+U^{2}+\cdots +U^{\tau -1}+U^{\tau }-U^{\tau })=\underset{\tau \to \infty }{\lim}U(I+U+U^{2}+\cdots +U^{\tau -1}+U^{\tau })-\underset{\tau \to \infty }{lim}UU^{\tau }=U{\left(I-U\right)}^{-1}-UO=U{\left(I-U\right)}^{-1}$$

and then there will be $[t_{ij}^{2}{]}_{n\times n}=V{\left(I-V\right)}^{-1}$. This completes the proof of Proposition 3.

Then the author will prove the elements of the *T* also are IFNs.

**Proposition 4** Let $t_{ij}=(t_{ij}^{1},t_{ij}^{2}),$ then $0\leq t_{ij}^{1}+t_{ij}^{2}\leq 1.$

***Proof*** By (13) and Proposition 1 there are 0≤*u*_*ij*_,*v*_*ij*_≤1 and *O*≤*U*,*V*≤*I*. Based on the matrix operation properties [68], there are (*I*−*U*)^−1^<*I* and (*I*−*V*)^−1^<*I*. By (18), ${[t}_{ij}^{1}{]}_{n\times n}$ and ${[t}_{ij}^{2}{]}_{n\times n}$ can be expressed by

$${\left[t_{ij}^{2}\right]}_{n\times n}=V{\left(I-V\right)}^{-1}\leq VI=[\begin{array}{cccc} \sum_{i=1}^{n}{\bar{v}}_{1i}/n & \sum_{i=1}^{n}{\bar{v}}_{1i}/n & \cdots & \sum_{i=1}^{n}{\bar{v}}_{1i}/n\\ \sum_{i=1}^{n}{\bar{v}}_{2i}/n & \sum_{i=1}^{n}{\bar{v}}_{2i}/n & \cdots & \sum_{i=1}^{n}{\bar{v}}_{2i}/n\\ \vdots & \vdots & \ddots & \vdots \\ \sum_{i=1}^{n}{\bar{v}}_{ni}/n & \sum_{i=1}^{n}{\bar{v}}_{ni}/n & \cdots & \sum_{i=1}^{n}{\bar{v}}_{ni}/n \end{array}].$$

Then there is ${[t}_{ij}^{1}{]}_{n\times n}+{[t}_{ij}^{2}{]}_{n\times n}\leq UI+VI\leq I$, where

$${\left[t_{ij}^{1}\right]}_{n\times n}=U{\left(I-U\right)}^{-1}\leq UI=[\begin{array}{cccc} \sum_{i=1}^{n}{\bar{u}}_{1i}/n & \sum_{i=1}^{n}{\bar{u}}_{1i}/n & \cdots & \sum_{i=1}^{n}{\bar{u}}_{1i}/n\\ \sum_{i=1}^{n}{\bar{u}}_{2i}/n & \sum_{i=1}^{n}{\bar{u}}_{2i}/n & \cdots & \sum_{i=1}^{n}{\bar{u}}_{2i}/n\\ \vdots & \vdots & \ddots & \vdots \\ \sum_{i=1}^{n}{\bar{u}}_{ni}/n & \sum_{i=1}^{n}{\bar{u}}_{ni}/n & \cdots & \sum_{i=1}^{n}{\bar{u}}_{ni}/n \end{array}],$$

$$UI+VI=[\begin{array}{ccc} (\sum_{i=1}^{n}{\bar{u}}_{1i}+\sum_{i=1}^{n}{\bar{v}}_{1i})/n & \cdots & (\sum_{i=1}^{n}{\bar{u}}_{1i}+\sum_{i=1}^{n}{\bar{v}}_{1i})/n\\ \vdots & \ddots & \vdots \\ (\sum_{i=1}^{n}{\bar{u}}_{ni}+\sum_{i=1}^{n}{\bar{v}}_{ni})/n & \cdots & (\sum_{i=1}^{n}{\bar{u}}_{ni}+\sum_{i=1}^{n}{\bar{v}}_{ni})/n \end{array}].$$

Thus, $t_{ij}=(t_{ij}^{1},t_{ij}^{2})$ are IFNs.

Let $c_{i}^{\prime }$ denote the overall degree that other factors are influenced by factor *F*_*i*_ and it can be derived by

$$c_{i}^{\prime }=t_{i1}⊕t_{i2}⊕\ldots ⊕t_{in},i=1,2,\ldots ,n.$$ (18)

Let $h_{i}^{\prime }$ be the overall degree that factor *F*_*i*_ is affected by others, which can be calculate by

$$h_{i}^{\prime }=t_{1i}⊕t_{2i}⊕\ldots ⊕t_{ni},i=1,2,\ldots ,n.$$ (19)

To get the ranking order of factor importance and the factor classification, the author applies function *S* for defuzzifying intuitionistic fuzzy numbers $c_{i}^{\prime }$ and $h_{i}^{\prime }$, i.e.,

$$c_{i}=c_{i}^{\prime 1}+\frac{1+c_{i}^{\prime 1}-c_{i}^{\prime 2}}{2}(1-c_{i}^{\prime 1}-c_{i}^{\prime 2}),i=1,2,\ldots ,n.$$ (20)

$$h_{i}=h_{i}^{\prime 1}+\frac{1+h_{i}^{\prime 1}-h_{i}^{\prime 2}}{2}(1-h_{i}^{\prime 1}-h_{i}^{\prime 2}),i=1,2,\ldots ,n.$$ (21)

The total effect both given and received by the *i*th factor can be obtained with (22), denoted as index *D*_*i*,_

$$D_{i}=c_{i}+h_{i},i=1,2,\ldots ,n.$$ (22)

And the author will get *R*_*i*_ be the relation of factor *F*_*i*_, representing the classification of the *i*^*th*^ factor as follows,

$$R_{i}=c_{i}-h_{i},i=1,2,\ldots ,n.$$ (23)

If *R*_*i*_≥0, then factor *F*_*i*_ is a cause factor. If *R*_*i*_≤0, then the *i*^*th*^ factor *F*_*i*_ is an effect factor [69].

The procedure of our proposed method is further concluded as below:

***Step 1*:** Build initial intuitionistic fuzzy direct-relation matrix $Z^{k}={\left[z_{ij}^{k}\right]}_{n\times n},k=1,2,\ldots ,m$.

***Step 2*:** Calculate group intuitionistic fuzzy direct-relation matrix $Z={\left[z_{ij}\right]}_{n\times n}$ using (5).

***Step 3*:** Calculate normalized intuitionistic fuzzy direct-relation matrix $Y={\left[y_{ij}\right]}_{n\times n}$ using (14).

***Step 4*:** Calculate intuitionistic fuzzy over-relation matrix $T={\left[t_{ij}\right]}_{n\times n}.$

***Step 5*:** Determine the intuitionistic fuzzy overall degree of influencing and influenced correlations of factor $F_{i},c_{i}^{\prime }$ and $h_{i}^{\prime },i=1,2,\ldots ,n$ using (18) and (19).

***Step 6*:** Determine the prominence of factors and the relationships between them, *D*_*i*_ and *R*_*i*_,*i* = 1,2,…,*n* using (22) and (23).

***Step 7*:** Construct the causal and effect diagram with *D*_*i*_ and *R*_*i*_,*i* = 1,2,…,*n*.

## 4 A real case of electric vehicles

In this section, a real case is used to demonstrate the potential applicability of the proposed method. In order to deal with petroleum dependence and environment pollution, particularly from a perspective of transportation sustainability, it is vital to improve newly emerging clean energy technology and promote adoption of EVs [70–72]. In addition, equipped with the intelligent electronic system, EVs also contribute significantly to smart logistics [73]. Although the number of EVs constitutes a minor fraction of the overall vehicle population, EV will rapidly grow and further develop in the coming years as the EV market is estimated to reach 700 million by 2040 [74].

However, the successful market penetration of EV is highly dependent on the development of its battery technology [75]. The improvement of EV battery technology usually has many uncertain factors of technical specifications such as power density, safety and security, and recharge. To identify the importance and classification of the factors of battery technology, an EV company conducted a factor analysis for promoting the development of EV battery technology using the extended DEMATEL method proposed in Section 3. In this section, the process and outcomes of the factor identification are provided.

A committee was formed with six experts: three came from academia and the other three from the industry, whose information is shown in Table 1. The committee jointly identified the possible factors that influence the development of EV battery technology. The committee then finalized the primary factors after a brainstorming meeting and an in-depth discussion. In the online brainstorming meeting, six experts leveraging their individual knowledge and industry experience, identified 5–7 factors of battery technology influencing EV adoption. After consolidating overlapping and dimensionally similar factors, a total of 15 distinct factors were derived. Each expert provided rationale and supporting evidence for the factors they proposed and challenged those they did not endorse. Subsequently, the experts re-ranked these 15 factors based on their significance, with the highest-ranked factor assigned 15 points, decreasing by 1 point for each subsequent rank. The top ten factors with the highest total scores were selected for this study (shown in Table 2). Factors that were excluded included battery compatibility, battery brand, transparency of battery performance, abuse tolerance of batteries, and after-sales service for batteries. These factors were primarily variables that were questioned during the discussion, as they were considered either too technical for customer awareness or infrequently mentioned in existing literature, lacking empirical support.

**Table 1:** Expert information.

Expert	Industry	Expertise	Experience (years)
***E***_***1***_	Academia	Electric vehicle operation management	10–15
***E***_***2***_	Academia	Innovation management	5–7
***E***_***3***_	Academia	Lithium battery-related materials	8–10
***E***_***4***_	Automobile	Design and integration of the vehicle system	6–9
***E***_***5***_	Automobile	Automotive marketing	5–8
***E***_***6***_	E-commerce	Operation and marketing of automotive components	7–9

**Table 2:** The factors of battery technology.

Factor	Description
Safety and security (*F*_1_)	The risk of sudden uncontrolled discharge in case of short-circuit, over-loading and overheating, e.g., spontaneous combustion or explosion.
Device and facility (*F*_2_)	Cost for installing the special device and facility to put energy into a secondary cell.
Recharge rate (*F*_3_)	Recharge time to make a dried battery to full.
Engineering robust (*F*_4_)	Making an EV operate efficiently in both hot summer and freezing winter conditions is a significant engineering challenge.
Specific energy (*F*_5_)	The amount of energy the battery can store per unit weight.
Specific power (*F*_6_)	The power-to-mass ratio, which represents the amount of power that batteries can deliver per kilogram of mass.
Life span (*F*_7_)	The average service life of the battery with the tolerance of charging and discharging.
Pollution (*F*_8_)	The pollution caused by the manufacture and remanufacture of the battery: carbon emissions, the toxicity of the material and so on.
Manufacture costs (*F*_9_)	The unit cost of the raw material for making a battery.
Material (*F*_10_)	Economy and convenience of raw materials for making batteries.

**Fig 2:**
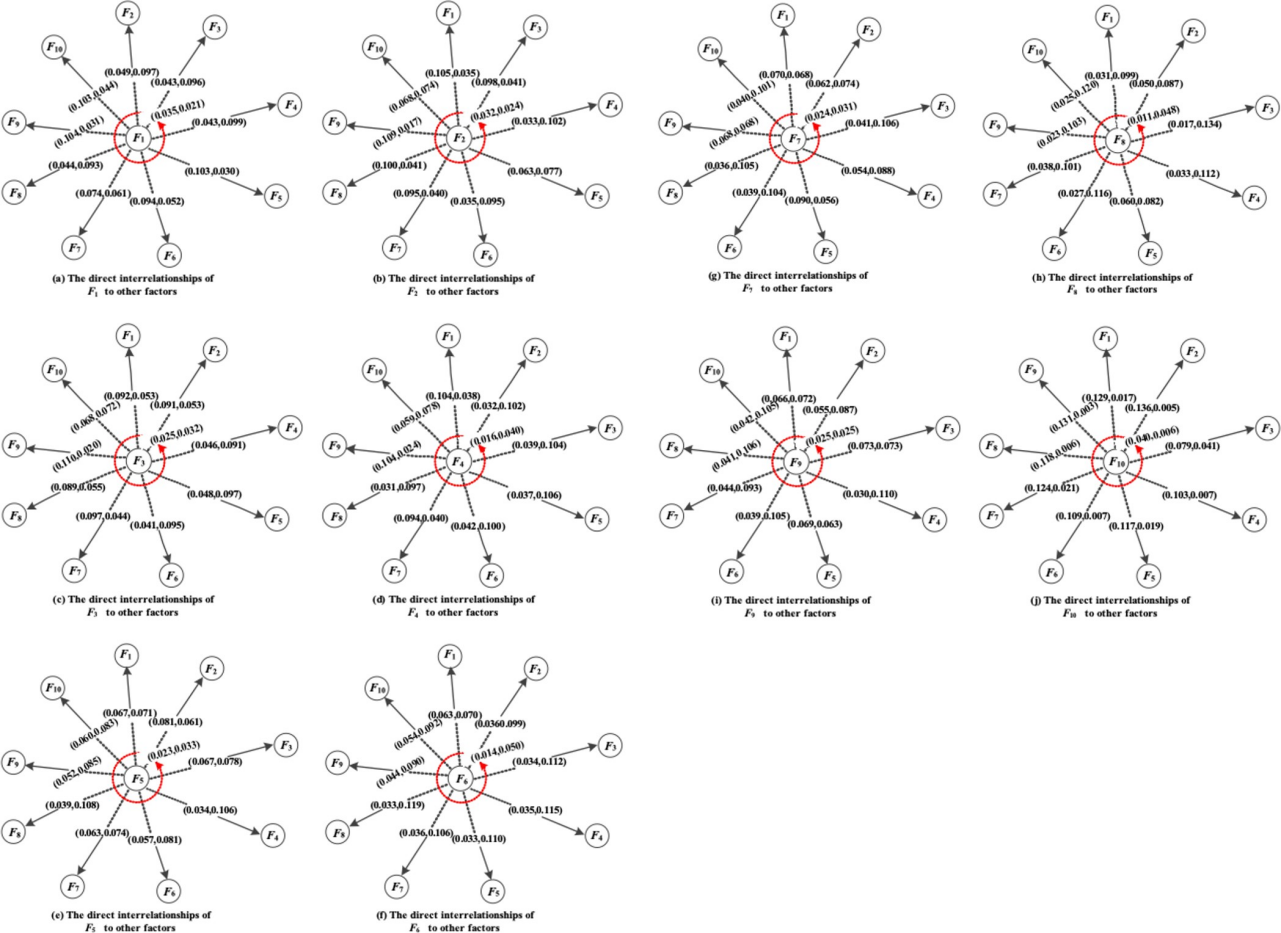
The direct interrelationships of each factor to other factors.

The six experts *E*_1_,*E*_2_,*E*_3_,*E*_4_,*E*_5_,*E*_6_ were tasked with offering their judgment on the existences and intensities of the factor correlations through a self-reported survey. By collecting the experts’ judgment on factors correlations, the author provides the initial intuitionistic fuzzy direct-relation matrix of the six experts using the nine linguistic rating scale (shown in Tables A1-A6 in S1 Appendix) [76]. Each set of data in the matrix represents the corresponding expert’s judgment of the relationship strength between two factors and is presented in the form of IFNs, in which (0, 0) indicates that there is no correlation between *F*_*i*_ and itself. Using (13), the experts’ individual matrices *Z*^1^,*Z*^2^,…,*Z*^*m*^ are aggregated into group intuitionistic fuzzy direct-relation matrix *Z* with *IFWA* as listed in Table 3. And using (14), matrix *Z* is normalized as the direct-relation intuitionistic fuzzy matrix *X* (presented in Table 4). Then, the over-relation matrix *T* in Table 5 is calculated with (18) and the over-relations of each factor with others are visualized in Fig 2 The figures show the overall degree of correlation between factors. For example, in Fig 2(A), the data on the arrows represent the degree of relationship between the factor *F*_1_ and the other factors, as indicated by IFNs. Furthermore, the overall correlations intensities of factor *F*_*i*_ to other and from other factors, i.e., $c_{i}^{\prime }$ and $h_{i}^{\prime }$ are obtained by (19) and (20) based on matrix *T*. The prominence and relation, *D*_*i*_ and *R*_*i*_, are be obtained by (23) and (24). The results are showed in Table 6.

**Table 3:** Group intuitionistic fuzzy direct-relation matrix *Z*.

	*F*_*1*_	*F*_*2*_	*F*_*3*_	*F*_*4*_	*F*_*5*_	*F*_*6*_	*F*_*7*_	*F*_*8*_	*F*_*9*_	*F*_*10*_
*F*_1_	(0.000,0.000)	(0.186,0.729)	(0.186,0.665)	(0.220,0.680)	(0.755,0.000)	(0.720,0.170)	(0.459,0.411)	(0.202,0.680)	(0.743,0.151)	(0.822,0.126)
*F*_2_	(0.738,0.182)	(0.000,0.000)	(0.771,0.126)	(0.119,0.780)	(0.300,0.541)	(0.102,0.729)	(0.654,0.204)	(0.767,0.135)	(0.755,0.000)	(0.423,0.515)
*F*_3_	(0.604,0.346)	(0.654,0.275)	(0.000,0.000)	(0.270,0.611)	(0.154,0.727)	(0.184,0.700)	(0.685,0.214)	(0.664,0.249)	(0.782,0.000)	(0.442,0.461)
*F*_4_	(0.809,0.126)	(0.100,0.765)	(0.202,0.713)	(0.000,0.000)	(0.086,0.763)	(0.220,0.663)	(0.730,0.126)	(0.135,0.682)	(0.782,0.000)	(0.384,0.442)
*F*_5_	(0.403,0.461)	(0.604,0.275)	(0.494,0.377)	(0.186,0.680)	(0.000,0.000)	(0.406,0.431)	(0.390,0.458)	(0.170,0.729)	(0.235,0.645)	(0.0403,0.461)
*F*_6_	(0.452,0.381)	(0.202.611)	(0.220,0.665)	(0.239,0.665)	(0.151,0.732)	(0.000,0.000)	(0.186,0.729)	(0.186,0.729)	(0.239,0.645)	(0.403,0.461)
*F*_7_	(0.452,0.419)	(0.421,0.415)	(0.220,0.682)	(0.421,0.475)	(0.712,0.214)	(0.220,0.680)	(0.000,0.000)	(0.186,0.716)	(0.435,0.464)	(0.204,0.657)
*F*_8_	(0.151,0.698)	(0.387,0.461)	(0.053,0.892)	(0.254,0.621)	(0.488,0.419)	(0.168,0.700)	(0.239,0.673)	(0.000,0.000)	(0.068,0.800)	(0.137,0.763)
*F*_9_	(0.435,0.461)	(0.352,0.528)	(0.585,0.300)	(0.168,0.700)	(0.510,0.285)	(0.235,0.665)	(0.222,0.638)	(0.237,0.700)	(0.000,0.000)	(0.235,0.682)
*F*_10_	(0.796,0.126)	(1.000,0.000)	(0.427,0.360)	(0.782,0.000)	(0.755,0.141)	(0.786,0.000)	(0.809,0.141)	(0.859,0.000)	(0.818,0.000)	(0.000,0.000)

**Table 4:** Normalized intuitionistic fuzzy direct-relation matrix *X*.

	*F*_1_	*F*_2_	*F*_3_	*F*_4_	*F*_5_	*F*_6_	*F*_7_	*F*_8_	*F*_9_	*F*_10_
*F*_1_	(0.000,0.000)	(0.019,0.073)	(0.019,0.066)	(0.022,0.068)	(0.076,0.000)	(0.072,0.017)	(0.046,0.041)	(0.020,0.068)	(0.074,0.015)	(0.082,0.013)
*F*_2_	(0.074,0.018)	(0.000,0.000)	(0.077,0.013)	(0.012,0.078)	(0.030,0.054)	(0.010,0.073)	(0.065,0.020)	(0.077,0.013)	(0.076,0.000)	(0.042,0.052)
*F*_3_	(0.060,0.035)	(0.065,0.027)	(0.000,0.000)	(0.027,0.061)	(0.015,0.073)	(0.018,0.070)	(0.069,0.021)	(0.066,0.025)	(0.078,0.000)	(0.044,0.046)
*F*_4_	(0.081,0.013)	(0.010,0.077)	(0.020,0.071)	(0.000,0.000)	(0.009,0.076)	(0.022,0.066)	(0.073,0.013)	(0.013,0.068)	(0.078,0.000)	(0.038,0.044)
*F*_5_	(0.040,0.046)	(0.060,0.027)	(0.049,0.038)	(0.019,0.068)	(0.000,0.000)	(0.041,0.043)	(0.039,0.046)	(0.017,0.073)	(0.023,0.065)	(0.040,0.046)
*F*_6_	(0.045,0.038)	(0.020.061)	(0.022,0.066)	(0.024,0.066)	(0.015,0.073)	(0.000,0.000)	(0.019,0.073)	(0.019,0.076)	(0.024,0.065)	(0.040,0.046)
*F*_7_	(0.045,0.042)	(0.042,0.042)	(0.022,0.068)	(0.042,0.048)	(0.071,0.021)	(0.022,0.068)	(0.000,0.000)	(0.019,0.072)	(0.044,0.046)	(0.020,0.066)
*F*_8_	(0.015,0.070)	(0.039,0.046)	(0.005,0.089)	(0.025,0.062)	(0.049,0.042)	(0.017,0.070)	(0.024,0.067)	(0.000,0.000)	(0.007,0.080)	(0.014,0.076)
*F*_9_	(0.044,0.046)	(0.035,0.053)	(0.058,0.030)	(0.017,0.070)	(0.051,0.029)	(0.023,0.066)	(0.022,0.064)	(0.024,0.070)	(0.000,0.000)	(0.023,0.068)
*F*_10_	(0.080,0.013)	(0.100,0.000)	(0.043,0.036)	(0.078,0.000)	(0.076,0.014)	(0.079,0.000)	(0.081,0.014)	(0.086,0.000)	(0.082,0.000)	(0.000,0.000)

**Table 5:** Intuitionistic fuzzy over-relation matrix *T*.

	*F*_1_	*F*_2_	*F*_3_	*F*_4_	*F*_5_	*F*_6_	*F*_7_	*F*_8_	*F*_9_	*F*_10_
*F*_1_	(0.035,0.021)	(0.049,0.097)	(0.043,0.096)	(0.043,0.099)	(0.103,0.030)	(0.094,0.052)	(0.074,0.061)	(0.044,0.093)	(0.104,0.031)	(0.103,0.044)
*F*_2_	(0.105,0.035)	(0.032,0.024)	(0.098,0.041)	(0.033,0.102)	(0.063,0.077)	(0.035,0.095)	(0.095,0.040)	(0.100,0.041)	(0.109,0.017)	(0.068,0.074)
*F*_3_	(0.092,0.053)	(0.091,0.053)	(0.025,0.032)	(0.046,0.091)	(0.048,0.097)	(0.041,0.095)	(0.097,0.044)	(0.089,0.055)	(0.110,0.020)	(0.068,0.072)
*F*_4_	(0.104,0.038)	(0.032,0.102)	(0.039,0.104)	(0.016,0.040)	(0.037,0.106)	(0.042,0.100)	(0.094,0.040)	(0.031,0.097)	(0.104,0.024)	(0.059,0.078)
*F*_5_	(0.067,0.071)	(0.081,0.061)	(0.067,0.078)	(0.034,0.106)	(0.023,0.033)	(0.057,0.081)	(0.063,0.074)	(0.039,0.108)	(0.052,0.085)	(0.060,0.083)
*F*_6_	(0.063,0.070)	(0.0360.099)	(0.034,0.112)	(0.035,0.115)	(0.033,0.110)	(0.014,0.050)	(0.036,0.106)	(0.033,0.119)	(0.044,0.090)	(0.054,0.092)
*F*_7_	(0.070,0.068)	(0.062,0.074)	(0.041,0.106)	(0.054,0.088)	(0.090,0.056)	(0.039,0.104)	(0.024,0.031)	(0.036,0.105)	(0.068,0.068)	(0.040,0.101)
*F*_8_	(0.031,0.099)	(0.050,0.087)	(0.017,0.134)	(0.033,0.112)	(0.060,0.082)	(0.027,0.116)	(0.038,0.101)	(0.011,0.048)	(0.023,0.103)	(0.025,0.120)
*F*_9_	(0.066,0.072)	(0.055,0.087)	(0.073,0.073)	(0.030,0.110)	(0.069,0.063)	(0.039,0.105)	(0.044,0.093)	(0.041,0.106)	(0.025,0.025)	(0.042,0.105)
*F*_10_	(0.129,0.017)	(0.136,0.005)	(0.079,0.041)	(0.103,0.007)	(0.117,0.019)	(0.109,0.007)	(0.124,0.021)	(0.118,0.006)	(0.131,0.003)	(0.040,0.006)

**Table 6:** Computational results.

	*c*_*i*_ *’*	*h*_*i*_ *’*	*c*_*i*_	*h*_*i*_	*D*_*i*_	*R*_*i*_
*F*_1_	(0.514,0.000)	(0.579,0.000)	0.881	0.886	1.768	-0.004
*F*_2_	(0.537,0.000)	(0.495,0.000)	0.887	0.894	1.747	0.038
*F*_3_	(0.522,0.000)	(0.471,0.000)	0.836	0.860	1.746	0.026
*F*_4_	(0.440,0.000)	(0.377,0.000)	0.818	0.806	1.649	0.037
*F*_5_	(0.429,0.000)	(0.521,0.000)	0.872	0.885	1.722	-0.048
*F*_6_	(0.323,0.000)	(0.422,0.000)	0.839	0.833	1.604	-0.062
*F*_7_	(0.418,0.000)	(0.559,0.000)	0.878	0.903	1.733	-0.072
*F*_8_	(0.275,0.000)	(0.488,0.000)	0.689	0.869	1.606	-0.132
*F*_9_	(0.393,0.000)	(0.603,0.000)	0.836	0.921	1.737	-0.105
*F*_10_	(0.685,0.000)	(0.477,0.000)	0.919	0.863	1.813	0.087

**Fig 3:**
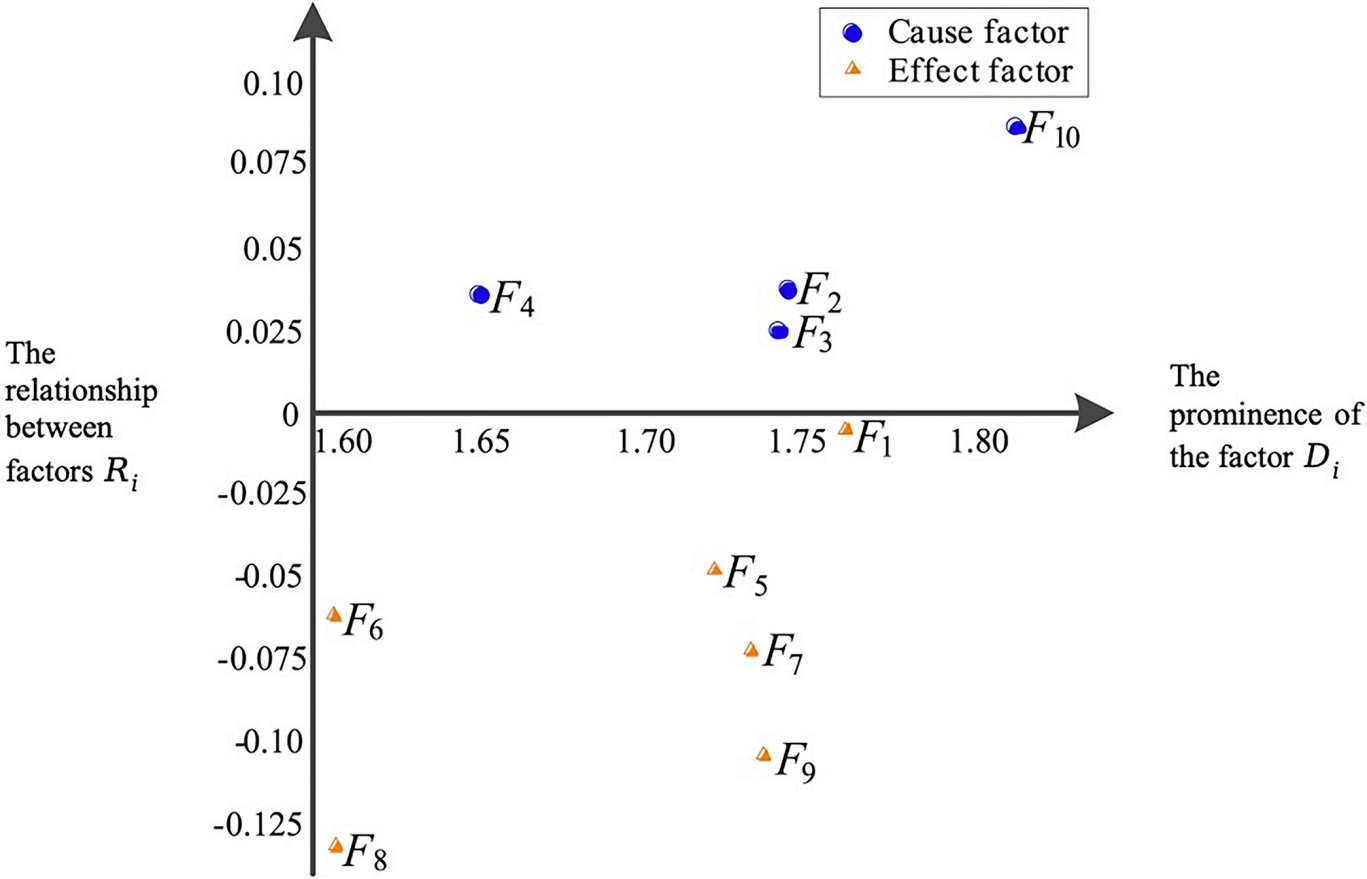
The causal and effect diagram.

A causal and effect diagram is provided to profusely show the factor prominence and relations in Fig 3. The factors *F*_2_,*F*_3_,*F*_4_ and *F*_10_ over horizontal axis *R* are cause factors, while the rest are considered effect factors. The importance ranking of the ten factors can be obtained with the factor prominence, i.e., $F_{10}≻F_{1}≻F_{2}≻F_{3}≻F_{9}≻F_{7}≻F_{5}≻F_{4}≻F_{8}≻F_{6}$. Thus, *F*_10_ is the most important factor for the development of EV battery technology and is desired to draw more attention.

## 5 Discussion

In this section, the results obtained the section 4 is further discussed.

### 5.1 Cause factors of battery technology

In the case analysis, the cause factors obtained include *F*_2_, *F*_3_, *F*_4_, and *F*_10_. *F*_10_ is the most critical of all factors, a finding that is consistent with the research conclusions of Prakash et al. (2018) regarding the accessibility of battery materials, which is considered an important factor in promoting the EV adoption [77]. Furthermore, the relationships between *F*_10_ and other factors are also noteworthy. As can be seen from Fig 3, there is a strong correlation between *F*_10_ and *F*_1_, *F*_2_, *F*_7_, and *F*_9_. In practice, battery materials indeed largely determine the performance and cost of batteries, hence the findings of this study are also consistent with practical phenomena. *F*_2_ is the next critical cause factor. Charging facilities, as complementary goods to batteries, affect the usefulness and ease of use of EVs and have been identified as key influencing factors in existing research on EV adoption [78, 79]. The installation cost of charging facilities, whether public or private, is a key consideration for installers and impacts the expansion of the charging network. The strong correlation between *F*_2_ and *F*_1_ and *F*_9_ also suggests that customers consider charging facilities in conjunction with batteries in terms of safety and cost, further reflecting the importance of charging facilities for EV adoption. *F*_3_ is the key factor following *F*_2_. This factor is one of the important manifestations of battery performance, a parameter that customers pay attention to when purchasing EVs, and has been proven to be an important influencing factor in existing EV adoption literature [80, 81]. *F*_3_ is strongly correlated with *F*_9_, where the former affects the time cost of using EVs, while the latter influences the purchase cost. It can be seen that customers consider costs in multiple dimensions when adopting EVs. *F*_4_ is another important performance indicator of batteries. Although rarely mentioned in existing EV adoption research, it is a highly focused aspect in battery technology research [82, 83]. The temperature management of batteries will affect the battery efficiency and the driving range of EVs, especially under extreme conditions. And driving range is one of the key factors influencing EVs [84, 85].

### 5.2 Effect factors of battery technology

The effect factors obtained in Section 4 can be classified into three categories. *F*_5_ and *F*_6_ constitute the first category, which are the key technical parameters of the vehicle battery. These two factors reflect the battery efficiency and determine the energy consumption and driving range of EVs to a certain extent, which are pivotal considerations in consumers’ EV purchase decision-making process [84, 86, 87]. In terms of factor interrelations, these two factors are predominantly influenced by the cause factor *F*_10_. The second category encompasses *F*_1_, *F*_7_ and *F*_9_. Beyond being attributes of battery technology, they also represent the safety, product lifespan, and cost of EVs. These factors have been demonstrated in multiple studies to be critical in influencing the EV adoption [88–90]. This group of factors is influenced by the most cause factors, indicating that there are diverse avenues to enhance customers’ perceptions regarding battery safety, lifespan, and cost. The third category consists of *F*_8_. As a green product, while EVs inherently possess an advantage in terms of exhaust emissions, the potential pollution associated with battery production and recycling is also a concern for consumers, particularly those considering EVs for environmental reasons. *F*_8_ is primarily influenced by *F*_10_ and *F*_2_. The association between *F*_8_ and *F*_2_ yields intriguing results, suggesting that the operational cost of the battery may mitigate consumers’ concerns about battery pollution or even enhance their tolerance for it. Similar conclusions exist in EV adoption research, such as the finding that improved charging convenience can increase consumers’ price tolerance [91].

### 5.3 Practical implications

All the aforementioned cause and effect factors are related to the key attributes of EVs, and the EV attributes (such as range and cost) have been confirmed by existing literature to be related to customer adoption willingness. This indicates that the results of this case analysis have a certain theoretical basis and to some extent verify the accuracy of the method proposed in this study. In addition, the case analysis further maps the attribute factors affecting EV adoption to the corresponding battery technology factors, providing more specific and detailed insights for enterprises when conducting battery technology research and development. The practical implications of the aforementioned findings can be explored from three perspectives.

First, there should be an increased focus on the impact of cause factors on battery technology and EV adoption. Cause factors exert not only a direct effect on outcomes but also influence other effect factors indirectly. For instance, batteries encompass a range of battery chemistries that utilize different combinations of anode and cathode materials, each exhibiting unique features in terms of safety, performance, cost, and other aspects. Consequently, EV companies could experiment with various combinations to mitigate the impact of the most critical factor *F*_10_. Second, it is essential to harness the feedback provided by effect factors. Although effect factors require the impetus of cause factors to exert their efforts, this dependency can be leveraged to assess the improvement measures targeting cause factors or to identify cause factors in need of enhancement through the evaluation of effect factors. For instance, as categorized in Section 5.2, the battery technology of EVs can be assessed along three dimensions: battery attributes performance, energy efficiency, and environmental performance. Once a specific area of weakness is identified (e.g., *F*_8_, representing environmental performance), the underlying cause factors (*F*_2_ and *F*_10_) can be targeted for improvement to address the technological shortcomings of the battery. Measures such as enhancing the convenience and economic viability of battery use or refining the production and recycling models of batteries are suggested in the case involving *F*_8_. Last but not least, attention should be given to the relationship between cause factors and effect factors. For example, by improving the performance of charging infrastructure or reducing installation costs, companies may compensate for issues related to battery efficiency, thereby enhancing customers’ perception of the usefulness and ease of use of batteries, and even their tolerance for potential battery pollution. In addition, targeted strategies can be implemented to alleviate the negative or indeterminate impacts arising from cause factors. For instance, EV companies may undertake an array of battery safety tests under various conditions and investigate novel materials to augment battery security. Such proactive measures are designed to diminish the uncertain influence of factor *F*_10_ on effect factor *F*_1_.

## 6 Conclusion

In real-world contexts, decision-makers typically express their judgment or opinions that are fuzzy and uncertain due to a variety of factors such as their limited expertise or required information, time pressure. Given this scenario, utilizing intuitionistic fuzzy information presents a viable approach for providing prescriptive solutions to the predicament of imprecise or uncertain judgment of decision-makers. Essentially, intuitionistic fuzzy information constitutes a significant type of input data integral to the intricate analysis of interdependent factors due to the complex transit relations and high uncertainty influences among factors. However, there is a paucity of an in-depth investigation on the dependent factor analysis methods using intuitionistic fuzzy information. To extend this stream of research and fill the research gap, this study attempts to usher and incorporate intuitionistic fuzzy information in the dependent factor analysis method. A real case of EVs is utilized to exemplify the principles and procedure of the proposed method. Through this case analysis, four cause factors and six effect factors that cover the safety, performance and cost of EV battery technology are identified, which helps EV company implement certain measures to promote the development of EV battery.

The DEMATEL method, by integrating quantitative data with the qualitative judgments of experts, offers a more comprehensive and flexible analytical framework. It is particularly applicable to fields involving complex interdependencies and high uncertainty, such as risk management, supply chain optimization, environmental impact assessment, and medical decision support, thereby enhancing the quality and reliability of decision-making. Scholars have also improved and expanded the method for various application scenarios, with a focus on addressing the issues of expert-expressed fuzziness and insufficient information. Improvements in this direction have led to the development of extended DEMATEL methods, such as grey-DEMATEL, fuzzy DEMATEL methods with 2-tuple linguistics, Z-numbers, and T-spherical fuzzy sets [14, 37, 38, 41]. As the DEMATEL method continues to evolve, it has been applied to emerging fields like smart supply chains, blockchain technology applications, and smart cities [92–94]. This paper also proposes a paradigmatic extension of the DEMATEL method in response to the intuitive fuzziness expressed by experts, employing intuitionistic information to analyze the interrelationships among factors. This expanded method makes a substantial contribution to scholarly research by pioneering a pathway for researchers to delve deeper into assessing the significance and categorization of individual factors. Additionally, it aids decision-makers in conducting factor analysis within the domains of risk and operational management. This new method assumes vital importance because it allocates efforts to addressing the gaps of the existing DEMATEL methods, thus significantly enriching the theoretical foundation of the DEMATEL method. In particular, this new method sheds new light on several glaring improvements in comparison with the extant studies in this research domain.

First, the proposed extended DEMATEL method is well-suited for addressing the analysis of dependent factors within an intuitionistic fuzzy context, where the decision analytic situation can be so uncertain that decision-makers can resort to their experience and knowledge to provide intuitionistic fuzzy judgment.

Second, the proposed method is capable of processing the intuitionistic fuzzy information on the correlations among factors, which might be embedded with the features encompassing affirmation, negation, and hesitation of the decision-makers’ cognitions. The author believes that this methodological advancement will overcome the limitation of the existing DEMATEL methods in terms of dependent factor analysis which could suffer from the loss of the initial judgment information.

Third, the proposed method sheds light on developing specific methodologies for processing intuitionistic fuzzy correlations among dependent factors. In light of this methodological extension, the author proposes primary propositions and lemmas to transform and aggregate the correlation relation matrixes in intuitionistic fuzzy numbers. Based on these key theoretical techniques, comprehensive principles and procedures are formulated for addressing issues related to the analysis of interdependent factors using intuitionistic fuzzy information.

Despite the comparative advancements of this proposed method, this study inevitably has several limitations. On the one hand, this study assumes that all the decision-makers have identical weight, but in fact they may hold different weights because different decision-makers may possess different levels of knowledge and experiences. On the other hand, all the factors are operationalized as quantitative attributes in this study. Yet it is also feasible that scholars may resolve the problems that involve more complex information of hybrid factor correlations, such as linguistic terms and interval intuitionistic fuzzy numbers. Additionally, to better demonstrate the application of the proposed method and focus on the impact of key variables, the number of analysis variables in this case study was controlled. Yet, as the application scenarios of the DEMATEL method change, many issues will involve an increasing number of factors. This complexity necessitates the development of effective algorithms and computational tools to manage the calculation and analysis process. Interpreting the results of large-scale variable analysis is also challenging, and the visualization of results and the ability to mine useful information are areas that require further exploration in the future. Given the potential for future research, the author thus calls for additional investigations and analyses in this field of study to further consolidate this paradigm.

## Supporting information

S1 Appendix Tables A1-A6 are the initial intuitionistic fuzzy direct-relation matrix of the six experts. (DOCX)

S1 File Minimal data set for the research. (DOCX)
