# Supplementary material for: Extended DEMATEL method with intuitionistic fuzzy information: A case of electric vehicles
Source: PLoS One. 2024 Dec 19;19(12):e0314650. doi: 10.1371/journal.pone.0314650 (PMC11658640; doi:10.1371/journal.pone.0314650)
Supplement: S1 Appendix — (DOCX) [file pone.0314650.s001.docx]

# Appendix

Table A1-A6 are the initial intuitionistic fuzzy direct-relation matrix of the six experts.

**Table A1. Initial intuitionistic fuzzy direct-relation matrix provided by *E*_1_.**

|  | ***F*_1_** | ***F*_2_** | ***F*_3_** | ***F*_4_** | ***F*_5_** | ***F*_6_** | ***F*_7_** | ***F*_8_** | ***F*_9_** | ***F*_10_** |
| --- | --- | --- | --- | --- | --- | --- | --- | --- | --- | --- |
| *F*_1_ | (0.0,0.0) | (0.2,0.7) | (0.3,0.6.) | (0.2,0.7) | (0.8,0.1) | (0.8,0.1) | (0.5,0.5) | (0.2,0.7) | (0.8,0.1) | (0.9,0.1) |
| *F*_2_ | (0.7,0.2) | (0.0,0.0) | (0.8,0.1) | (0.0,0.9) | (0.1,0.8) | (0.0,0.8) | (0.7,0.1) | (0.8,0.1) | (0.8,0.0) | (0.5,0.5) |
| *F*_3_ | (0.6,0.3) | (0.7,0.2) | (0.0,0.0) | (0.3,0.6) | (0.2,0.7) | (0.2,0.7) | (0.7,0.2) | (0.7,0.2) | (0.7,0.2) | (0.4,0.4) |
| *F*_4_ | (0.8,0.2) | (0.1,0.8) | (0.2,0.7) | (0.0,0.0) | (0.0,0.8) | (0.5,0.5) | (0.4,0.4) | (0.1,0.8) | (0.2,0.7) | (0.4,0.5) |
| *F*_5_ | (0.4,0.4) | (0.6,0.2) | (0.5,0.5) | (0.2,0.7) | (0.0,0.0) | (0.5,0.5) | (0.4,0.4) | (0.1,0.8) | (0.2,0.7) | (0.4,0.5) |
| *F*_6_ | (0.4,0.4) | (0.2,0.6) | (0.2,0.7) | (0.2,0.7) | (0.2,0.7) | (0.0,0.0) | (0.1,0.8) | (0.1,0.8) | (0.2,0.7) | (0.4,0.5) |
| *F_7_* | (0.4,0.6) | (0.4,0.4) | (0.2,0.7) | (0.5,0.5) | (0.6,0.2) | (0.2,0.7) | (0.0,0.0) | (0.2,0.7) | (0.5,0.5) | (0.1,0.8) |
| *F*_8_ | (0.1,0.7) | (0.4,0.4) | (0.0,1.0) | (0.2,0.7) | (0.5,0.5) | (0.1,0.7) | (0.2,0.7) | (0.0,0.0) | (0.1,0.8) | (0.1,0.8) |
| *F*_9_ | (0.5,0.5) | (0.3,0.6) | (0.6,0.3) | (0.1,0.7) | (0.4,0.5) | (0.3,0.7) | (0.2,0.7) | (0.2,0.7) | (0.0,0.0) | (0.2,0.7) |
| *F*_10_ | (0.8,0.1) | (1.0,0.0) | (0.6,0.3) | (0.8,0.1) | (0.8,0.2) | (0.8,0.1) | (0.8,0.1) | (0.9,0.1) | (0.9,0.1) | (0.0,0.0) |

**Table A2. Initial intuitionistic fuzzy direct-relation matrix provided by *E*_2_.**

|  | ***F*_1_** | ***F*_2_** | ***F*_3_** | ***F*_4_** | ***F*_5_** | ***F*_6_** | ***F*_7_** | ***F*_8_** | ***F*_9_** | ***F*_10_** |
| --- | --- | --- | --- | --- | --- | --- | --- | --- | --- | --- |
| *F*_1_ | (0.0,0.0) | (0.2,0.8) | (0.2,0.7) | (0.2,0.7) | (0.7,0.1) | (0.7,0.2) | (0.6,0.2) | (0.2,0.7) | (0.7,0.2) | (0.8,0.1) |
| *F*_2_ | (0.7,0.2) | (0.0,0.0) | (0.8,0.1) | (0.1,0.8) | (0.1,0.7) | (0.2,0.6) | (0.7,0.2) | (0.8,0.1) | (0.7,0.2) | (0.3,0.5) |
| *F*_3_ | (0.6,0.4) | (0.7,0.2) | (0.0,0.0) | (0.3,0.6) | (0.1,0.9) | (0.2,0.7) | (0.7,0.2) | (0.6,0.3) | (0.8,0.0) | (0.6,0.4) |
| *F*_4_ | (0.8,0.1) | (0.1,0.7) | (0.1,0.8) | (0.0,0.0) | (0.0,0.8) | (0.3,0.6) | (0.8,0.1) | (0.1,0.7) | (0.8,0.1) | (0.4,0.5) |
| *F*_5_ | (0.3,0.6) | (0.6,0.3) | (0.4,0.4) | (0.3,0.6) | (0.0,0.0) | (0.4,0.4) | (0.5,0.4) | (0.3,0.6) | (0.3,0.7) | (0.3,0.4) |
| *F*_6_ | (0.5,0.3) | (0.2,0.6) | (0.3,0.6) | (0.2,0.7) | (0.1,0.7) | (0.0,0.0) | (0.2,0.8) | (0.1,0.9) | (0.2,0.7) | (0.3,0.4) |
| *F*_7_ | (0.5,0.5) | (0.4,0.4) | (0.1,0.7) | (0.4,0.4) | (0.7,0.2) | (0.3,0.7) | (0.0,0.0) | (0.1,0.7) | (0.5,0.4) | (0.2,0.6) |
| *F*_8_ | (0.1,0.7) | (0.3,0.6) | (0.0,1.0) | (0.2,0.7) | (0.5,0.4) | (0.2,0.7) | (0.4,0.5) | (0.0,0.0) | (0.1,0.8) | (0.1,0.8) |
| *F*_9_ | (0.4,0.6) | (0.4,0.4) | (0.5,0.3) | (0.2,0.7) | (0.4,0.4) | (0.3,0.6) | (0.4,0.4) | (0.3,0.7) | (0.0,0.0) | (0.2,0.7) |
| *F*_10_ | (0.7,0.2) | (0.9,0.0) | (0.3,0.5) | (0.9,0.0) | (0.7,0.2) | (0.7,0.1) | (0.9,0.1) | (0.8,0.1) | (0.8,0.1) | (0.0,0.0) |

**Table A3. Initial intuitionistic fuzzy direct-relation matrix provided by *E*_3_.**

|  | ***F*_1_** | ***F*_2_** | ***F*_3_** | ***F*_4_** | ***F*_5_** | ***F*_6_** | ***F*_7_** | ***F*_8_** | ***F*_9_** | ***F*_10_** |
| --- | --- | --- | --- | --- | --- | --- | --- | --- | --- | --- |
| *F*_1_ | (0.0,0.0) | (0.1,0.8) | (0.2,0.7) | (0.3,0.6) | (0.8,0) | (0.7,0.3) | (0.4,0.4) | (0.1,0.8) | (0.8,0.1) | (0.8,0.1) |
| *F*_2_ | (0.7,0.3) | (0.0,0.0) | (0.8,0.2) | (0.2,0.7) | (0.1,0.7) | (0.1,0.8) | (0.6,0.3) | (0.8,0.1) | (0.7,0.2) | (0.5,0.5) |
| *F*_3_ | (0.6,0.4) | (0.6,0.4) | (0.0,0.0) | (0.3,0.6) | (0.3,0.6) | (0.2,0.7) | (0.6,0.3) | (0.8,0.1) | (0.7,0.1) | (0.5,0.5) |
| *F*_4_ | (0.7,0.2) | (0.1,0.8) | (0.2,0.8) | (0.0,0.0) | (0.1,0.8) | (0.2,0.8) | (0.8,0.1) | (0.2,0.7) | (0.9,0.0) | (0.4,0.5) |
| *F*_5_ | (0.4,0.5) | (0.6,0.3) | (0.6,0.4) | (0.1,0.7) | (0.0,0.0) | (0.5,0.4) | (0.3,0.4) | (0.1,0.8) | (0.2,0.6) | (0.4,0.4) |
| *F*_6_ | (0.4,0.4) | (0.2,0.6) | (0.2,0.7) | (0.3,0.6) | (0.2,0.7) | (0.0,0.0) | (0.3,0.6) | (0.2,0.7) | (0.3,0.6) | (0.4,0.4) |
| *F*_7_ | (0.4,0.4) | (0.5,0.4) | (0.2,0.7) | (0.4,0.6) | (0.8,0.2) | (0.1,0.8) | (0.0,0.0) | (0.2,0.7) | (0.4,0.5) | (0.3,0.5) |
| *F*_8_ | (0.2,0.7) | (0.5,0.5) | (0.0,1.0) | (0.2,0.7) | (0.5,0.5) | (0.2,0.7) | (0.3,0.7) | (0.0,0.0) | (0.1,0.8) | (0.1,0.8) |
| *F*_9_ | (0.4,0.5) | (0.4,0.5) | (0.6,0.3) | (0.2,0.7) | (0.6,0.3) | (0.2,0.6) | (0.2,0.7) | (0.3,0.7) | (0.0,0.0) | (0.3,0.6) |
| *F*_10_ | (0.9,0.1) | (0.8,0.1) | (0.4,0.4) | (0.7,0.2) | (0.8,0.1) | (0.8,0.2) | (0.8,0.2) | (0.9,0.0) | (0.7,0.2) | (0.0,0.0) |

**Table A4. Initial intuitionistic fuzzy direct-relation matrix provided by *E*_4_.**

|  | ***F*_1_** | ***F*_2_** | ***F*_3_** | ***F*_4_** | ***F*_5_** | ***F*_6_** | ***F*_7_** | ***F*_8_** | ***F*_9_** | ***F*_10_** |
| --- | --- | --- | --- | --- | --- | --- | --- | --- | --- | --- |
| *F*_1_ | (0.0,0.0) | (0.3,0.6) | (0.1,0.6) | (0.1,0.8) | (0.8,0.1) | (0.7,0.1) | (0.3,0.6) | (0.2,0.7) | (0.7,0.2) | (0.8,0.2) |
| *F*_2_ | (0.8,0.1) | (0.0,0.0) | (0.7,0.2) | (0.2,0.7) | (0.1,0.8) | (0.1,0.8) | (0.7,0.2) | (0.8,0.2) | (0.7,0.1) | (0.3,0.5) |
| *F*_3_ | (0.7,0.3) | (0.6,0.3) | (0.0,0.0) | (0.3,0.5) | (0.1,0.7) | (0.2,0.7) | (0.7,0.2) | (0.7,0.2) | (0.8,0.1) | (0.4,0.4) |
| *F4* | (0.9,0.1) | (0.1,0.7) | (0.2,0.7) | (0.0,0.0) | (0.2,0.6) | (0.3,0.6) | (0.6,0.2) | (0.1,0.7) | (0.8,0.1) | (0.3,0.5) |
| *F*_5_ | (0.4,0.4) | (0.6,0.2) | (0.5,0.4) | (0.2,0.6) | (0.0,0.0) | (0.3,0.5) | (0.3,0.6) | (0.2,0.7) | (0.2,0.7) | (0.4,0.6) |
| *F*_6_ | (0.5,0.4) | (0.3,0.5) | (0.3,0.6) | (0.2,0.7) | (0.1,0.8) | (0.0,0.0) | (0.2,0.7) | (0.3,0.7) | (0.4,0.5) | (0.4,0.6) |
| *F*_7_ | (0.5,0.3) | (0.4,0.4) | (0.3,0.7) | (0.4,0.4) | (0.7,0.2) | (0.3,0.6) | (0.0,0.0) | (0.3,0.7) | (0.4,0.4) | (0.2,0.7) |
| *F*_8_ | (0.2,0.7) | (0.4,0.4) | (0.2,0.7) | (0.2,0.7) | (0.4,0.6) | (0.1,0.7) | (0.1,0.9) | (0.0,0.0) | (0.0,0.8) | (0.3,0.6) |
| *F*_9_ | (0.4,0.4) | (0.3,0.5) | (0.6,0.3) | (0.2,0.7) | (0.4,0.3) | (0.2,0.7) | (0.2,0.7) | (0.2,0.7) | (0.0,0.0) | (0.2,0.7) |
| *F*_10_ | (0.8,0.1) | (1.0,0.0) | (0.4,0.3) | (0.7,0.2) | (0.7,0.1) | (0.6,0.2) | (0.8,0.2) | (0.8,0.2) | (0.7,0.2) | (0.0,0.0) |

**Table A5. Initial intuitionistic fuzzy direct-relation matrix provided by *E*_5._**

|  | ***F*_1_** | ***F*_2_** | ***F*_3_** | ***F*_4_** | ***F*_5_** | ***F*_6_** | ***F*_7_** | ***F*_8_** | ***F*_9_** | ***F*_10_** |
| --- | --- | --- | --- | --- | --- | --- | --- | --- | --- | --- |
| *F*_1_ | (0.0,0.0) | (0.2,0.7) | (0.1,0.7) | (0.2,0.7) | (0.7,0.1) | (0.7,0.2) | (0.4,0.4) | (0.3,0.6) | (0.8,0.1) | (0.8,0.1) |
| *F*_2_ | (0.7,0.3) | (0.0,0.0) | (0.7,0.1) | (0.1,0.8) | (0.1,0.8) | (0.1,0.7) | (0.6,0.3) | (0.8,0.1) | (0.8,0.1) | (0.4,0.6) |
| *F*_3_ | (0.5,0.4) | (0.7,0.3) | (0.0,0.0) | (0.3,0.6) | (0.1,0.8) | (0.1,0.7) | (0.7,0.2) | (0.5,0.5) | (0.7,0.2) | (0.3,0.6) |
| *F*_4_ | (0.8,0.1) | (0.1,0.8) | (0.3,0.7) | (0.0,0.0) | (0.1,0.8) | (0.1,0.7) | (0.6,0.2) | (0.1,0.7) | (0.7,0.1) | (0.4,0.3) |
| *F*_5_ | (0.5,0.5) | (0.7,0.3) | (0.3,0.3) | (0.2,0.7) | (0.0,0.0) | (0.4,0.4) | (0.3,0.6) | (0.2,0.7) | (0.3,0.5) | (0.5,0.5) |
| *F*_6_ | (0.5,0.4) | (0.2,0.6) | (0.1,0.7) | (0.4,0.6) | (0.2,0.8) | (0.0,0.0) | (0.2,0.7) | (0.2,0.7) | (0.1,0.7) | (0.5,0.5) |
| *F*_7_ | (0.4,0.3) | (0.5,0.4) | (0.2,0.7) | (0.3,0.6) | (0.6,0.3) | (0.2,0.6) | (0.0,0.0) | (0.2,0.7) | (0.4,0.5) | (0.3,0.6) |
| *F*_8_ | (0.2,0.6) | (0.4,0.5) | (0.0,0.8) | (0.4,0.4) | (0.6,0.3) | (0.2,0.7) | (0.2,0.6) | (0.0,0.0) | (0,0.8) | (0.1,0.8) |
| *F*_9_ | (0.5,0.4) | (0.4,0.6) | (0.6,0.3) | (0.2,0.7) | (0.6,0.1) | (0.2,0.7) | (0.2,0.7) | (0.3,0.7) | (0.0,0.0) | (0.3,0.7) |
| *F*_10_ | (0.7,0.2) | (0.7,0.2) | (0.3,0.3) | (0.7,0.1) | (0.8,0.1) | (0.8,0.2) | (0.7,0.2) | (0.9,0.0) | (0.9,0.0) | (0.0,0.0) |

**Table A6. Initial intuitionistic fuzzy direct-relation matrix provided by *E*_6_.**

|  | ***F*_1_** | ***F*_2_** | ***F*_3_** | ***F*_4_** | ***F*_5_** | ***F*_6_** | ***F*_7_** | ***F*_8_** | ***F*_9_** | ***F*_10_** |
| --- | --- | --- | --- | --- | --- | --- | --- | --- | --- | --- |
| *F*_1_ | (0.0,0.0) | (0.1,0.8) | (0.2,0.7) | (0.3,0.6) | (0.7,0.2) | (0.7,0.2) | (0.5,0.5) | (0.2,0.6) | (0.6,0.3) | (0.8,0.2) |
| *F*_2_ | (0.8,0.1) | (0.0,0.0) | (0.8,0.1) | (0.1,0.8) | (0.1,0.8) | (0.1,0.7) | (0.6,0.2) | (0.5,0.3) | (0.8,0.1) | (0.5,0.5) |
| *F*_3_ | (0.6,0.3) | (0.6,0.3) | (0.0,0.0) | (0.1,0.8) | (0.1,0.7) | (0.2,0.7) | (0.7,0.2) | (0.6,0.4) | (0.9,0.0) | (0.4,0.5) |
| *F*_4_ | (0.8,0.1) | (0.1,0.8) | (0.2,0.6) | (0.0,0.0) | (0.1,0.8) | (0.2,0.6) | (0.7,0.1) | (0.1,0.6) | (0.7,0.2) | (0.4,0.5) |
| *F*_5_ | (0.4,0.4) | (0.5,0.4) | (0.6,0.3) | (0.1,0.8) | (0.0,0.0) | (0.3,0.4) | (0.5,0.4) | (0.1,0.8) | (0.2,0.7) | (0.4,0.4) |
| *F*_6_ | (0.4,0.4) | (0.1,0.8) | (0.2,0.7) | (0.1,0.7) | (0.1,0.7) | (0.0,0.0) | (0.1,0.8) | (0.2,0.8) | (0.2,0.7) | (0.4,0.4) |
| *F*_7_ | (0.5,0.5) | (0.3,0.5) | (0.3,0.6) | (0.5,0.4) | (0.8,0.2) | (0.2,0.7) | (0.0,0.0) | (0.1,0.8) | (0.4,0.5) | (0.1,0.8) |
| *F*_8_ | (0.1,0.8) | (0.3,0.4) | (0.1,0.9) | (0.3,0.6) | (0.4,0.3) | (0.2,0.7) | (0.2,0.7) | (0.0,0.0) | (0.1,0.8) | (0.1,0.8) |
| *F*_9_ | (0.4,0.4) | (0.3,0.6) | (0.6,0.3) | (0.1,0.7) | (0.6,0.3) | (0.2,0.7) | (0.1,0.7) | (0.1,0.7) | (0.0,0.0) | (0.2,0.7) |
| *F*_10_ | (0.8,0.1) | (0.8,0.1) | (0.5,0.4) | (0.8,0.2) | (0.7,0.2) | (0.9,0.0) | (0.8,0.1) | (0.8,0.1) | (0.8,0.1) | (0.0,0.0) |
