# Supplementary material for: Extended DEMATEL method with intuitionistic fuzzy information: A case of electric vehicles
Source: PLoS One. 2024 Dec 19;19(12):e0314650. doi: 10.1371/journal.pone.0314650 (PMC11658640; doi:10.1371/journal.pone.0314650)
Supplement: S1 File — (DOCX) [file pone.0314650.s002.docx]

**Minimal data set for the research “Extended DEMATEL Method with Intuitionistic Fuzzy Information: A Case of Electric Vehicles”**

1. **The raw data collected from the experts.**

Table S1- Table S6 are the judgement data of the existences and intensities of the factor correlations, which are collected from the six experts through a self-reported survey.

**Table S1. Initial intuitionistic fuzzy direct-relation matrix provided by *E*_1_.**

|  | ***F*_1_** | ***F*_2_** | ***F*_3_** | ***F*_4_** | ***F*_5_** | ***F*_6_** | ***F*_7_** | ***F*_8_** | ***F*_9_** | ***F*_10_** |
| --- | --- | --- | --- | --- | --- | --- | --- | --- | --- | --- |
| *F*_1_ | (0.0,0.0) | (0.2,0.7) | (0.3,0.6.) | (0.2,0.7) | (0.8,0.1) | (0.8,0.1) | (0.5,0.5) | (0.2,0.7) | (0.8,0.1) | (0.9,0.1) |
| *F*_2_ | (0.7,0.2) | (0.0,0.0) | (0.8,0.1) | (0.0,0.9) | (0.1,0.8) | (0.0,0.8) | (0.7,0.1) | (0.8,0.1) | (0.8,0.0) | (0.5,0.5) |
| *F*_3_ | (0.6,0.3) | (0.7,0.2) | (0.0,0.0) | (0.3,0.6) | (0.2,0.7) | (0.2,0.7) | (0.7,0.2) | (0.7,0.2) | (0.7,0.2) | (0.4,0.4) |
| *F*_4_ | (0.8,0.2) | (0.1,0.8) | (0.2,0.7) | (0.0,0.0) | (0.0,0.8) | (0.5,0.5) | (0.4,0.4) | (0.1,0.8) | (0.2,0.7) | (0.4,0.5) |
| *F*_5_ | (0.4,0.4) | (0.6,0.2) | (0.5,0.5) | (0.2,0.7) | (0.0,0.0) | (0.5,0.5) | (0.4,0.4) | (0.1,0.8) | (0.2,0.7) | (0.4,0.5) |
| *F*_6_ | (0.4,0.4) | (0.2,0.6) | (0.2,0.7) | (0.2,0.7) | (0.2,0.7) | (0.0,0.0) | (0.1,0.8) | (0.1,0.8) | (0.2,0.7) | (0.4,0.5) |
| *F_7_* | (0.4,0.6) | (0.4,0.4) | (0.2,0.7) | (0.5,0.5) | (0.6,0.2) | (0.2,0.7) | (0.0,0.0) | (0.2,0.7) | (0.5,0.5) | (0.1,0.8) |
| *F*_8_ | (0.1,0.7) | (0.4,0.4) | (0.0,1.0) | (0.2,0.7) | (0.5,0.5) | (0.1,0.7) | (0.2,0.7) | (0.0,0.0) | (0.1,0.8) | (0.1,0.8) |
| *F*_9_ | (0.5,0.5) | (0.3,0.6) | (0.6,0.3) | (0.1,0.7) | (0.4,0.5) | (0.3,0.7) | (0.2,0.7) | (0.2,0.7) | (0.0,0.0) | (0.2,0.7) |
| *F*_10_ | (0.8,0.1) | (1.0,0.0) | (0.6,0.3) | (0.8,0.1) | (0.8,0.2) | (0.8,0.1) | (0.8,0.1) | (0.9,0.1) | (0.9,0.1) | (0.0,0.0) |

**Table S2. Initial intuitionistic fuzzy direct-relation matrix provided by *E*_2_.**

|  | ***F*_1_** | ***F*_2_** | ***F*_3_** | ***F*_4_** | ***F*_5_** | ***F*_6_** | ***F*_7_** | ***F*_8_** | ***F*_9_** | ***F*_10_** |
| --- | --- | --- | --- | --- | --- | --- | --- | --- | --- | --- |
| *F*_1_ | (0.0,0.0) | (0.2,0.8) | (0.2,0.7) | (0.2,0.7) | (0.7,0.1) | (0.7,0.2) | (0.6,0.2) | (0.2,0.7) | (0.7,0.2) | (0.8,0.1) |
| *F*_2_ | (0.7,0.2) | (0.0,0.0) | (0.8,0.1) | (0.1,0.8) | (0.1,0.7) | (0.2,0.6) | (0.7,0.2) | (0.8,0.1) | (0.7,0.2) | (0.3,0.5) |
| *F*_3_ | (0.6,0.4) | (0.7,0.2) | (0.0,0.0) | (0.3,0.6) | (0.1,0.9) | (0.2,0.7) | (0.7,0.2) | (0.6,0.3) | (0.8,0.0) | (0.6,0.4) |
| *F*_4_ | (0.8,0.1) | (0.1,0.7) | (0.1,0.8) | (0.0,0.0) | (0.0,0.8) | (0.3,0.6) | (0.8,0.1) | (0.1,0.7) | (0.8,0.1) | (0.4,0.5) |
| *F*_5_ | (0.3,0.6) | (0.6,0.3) | (0.4,0.4) | (0.3,0.6) | (0.0,0.0) | (0.4,0.4) | (0.5,0.4) | (0.3,0.6) | (0.3,0.7) | (0.3,0.4) |
| *F*_6_ | (0.5,0.3) | (0.2,0.6) | (0.3,0.6) | (0.2,0.7) | (0.1,0.7) | (0.0,0.0) | (0.2,0.8) | (0.1,0.9) | (0.2,0.7) | (0.3,0.4) |
| *F*_7_ | (0.5,0.5) | (0.4,0.4) | (0.1,0.7) | (0.4,0.4) | (0.7,0.2) | (0.3,0.7) | (0.0,0.0) | (0.1,0.7) | (0.5,0.4) | (0.2,0.6) |
| *F*_8_ | (0.1,0.7) | (0.3,0.6) | (0.0,1.0) | (0.2,0.7) | (0.5,0.4) | (0.2,0.7) | (0.4,0.5) | (0.0,0.0) | (0.1,0.8) | (0.1,0.8) |
| *F*_9_ | (0.4,0.6) | (0.4,0.4) | (0.5,0.3) | (0.2,0.7) | (0.4,0.4) | (0.3,0.6) | (0.4,0.4) | (0.3,0.7) | (0.0,0.0) | (0.2,0.7) |
| *F*_10_ | (0.7,0.2) | (0.9,0.0) | (0.3,0.5) | (0.9,0.0) | (0.7,0.2) | (0.7,0.1) | (0.9,0.1) | (0.8,0.1) | (0.8,0.1) | (0.0,0.0) |

**Table S3. Initial intuitionistic fuzzy direct-relation matrix provided by *E*_3_.**

|  | ***F*_1_** | ***F*_2_** | ***F*_3_** | ***F*_4_** | ***F*_5_** | ***F*_6_** | ***F*_7_** | ***F*_8_** | ***F*_9_** | ***F*_10_** |
| --- | --- | --- | --- | --- | --- | --- | --- | --- | --- | --- |
| *F*_1_ | (0.0,0.0) | (0.1,0.8) | (0.2,0.7) | (0.3,0.6) | (0.8,0) | (0.7,0.3) | (0.4,0.4) | (0.1,0.8) | (0.8,0.1) | (0.8,0.1) |
| *F*_2_ | (0.7,0.3) | (0.0,0.0) | (0.8,0.2) | (0.2,0.7) | (0.1,0.7) | (0.1,0.8) | (0.6,0.3) | (0.8,0.1) | (0.7,0.2) | (0.5,0.5) |
| *F*_3_ | (0.6,0.4) | (0.6,0.4) | (0.0,0.0) | (0.3,0.6) | (0.3,0.6) | (0.2,0.7) | (0.6,0.3) | (0.8,0.1) | (0.7,0.1) | (0.5,0.5) |
| *F*_4_ | (0.7,0.2) | (0.1,0.8) | (0.2,0.8) | (0.0,0.0) | (0.1,0.8) | (0.2,0.8) | (0.8,0.1) | (0.2,0.7) | (0.9,0.0) | (0.4,0.5) |
| *F*_5_ | (0.4,0.5) | (0.6,0.3) | (0.6,0.4) | (0.1,0.7) | (0.0,0.0) | (0.5,0.4) | (0.3,0.4) | (0.1,0.8) | (0.2,0.6) | (0.4,0.4) |
| *F*_6_ | (0.4,0.4) | (0.2,0.6) | (0.2,0.7) | (0.3,0.6) | (0.2,0.7) | (0.0,0.0) | (0.3,0.6) | (0.2,0.7) | (0.3,0.6) | (0.4,0.4) |
| *F*_7_ | (0.4,0.4) | (0.5,0.4) | (0.2,0.7) | (0.4,0.6) | (0.8,0.2) | (0.1,0.8) | (0.0,0.0) | (0.2,0.7) | (0.4,0.5) | (0.3,0.5) |
| *F*_8_ | (0.2,0.7) | (0.5,0.5) | (0.0,1.0) | (0.2,0.7) | (0.5,0.5) | (0.2,0.7) | (0.3,0.7) | (0.0,0.0) | (0.1,0.8) | (0.1,0.8) |
| *F*_9_ | (0.4,0.5) | (0.4,0.5) | (0.6,0.3) | (0.2,0.7) | (0.6,0.3) | (0.2,0.6) | (0.2,0.7) | (0.3,0.7) | (0.0,0.0) | (0.3,0.6) |
| *F*_10_ | (0.9,0.1) | (0.8,0.1) | (0.4,0.4) | (0.7,0.2) | (0.8,0.1) | (0.8,0.2) | (0.8,0.2) | (0.9,0.0) | (0.7,0.2) | (0.0,0.0) |

**Table S4. Initial intuitionistic fuzzy direct-relation matrix provided by *E*_4_.**

|  | ***F*_1_** | ***F*_2_** | ***F*_3_** | ***F*_4_** | ***F*_5_** | ***F*_6_** | ***F*_7_** | ***F*_8_** | ***F*_9_** | ***F*_10_** |
| --- | --- | --- | --- | --- | --- | --- | --- | --- | --- | --- |
| *F*_1_ | (0.0,0.0) | (0.3,0.6) | (0.1,0.6) | (0.1,0.8) | (0.8,0.1) | (0.7,0.1) | (0.3,0.6) | (0.2,0.7) | (0.7,0.2) | (0.8,0.2) |
| *F*_2_ | (0.8,0.1) | (0.0,0.0) | (0.7,0.2) | (0.2,0.7) | (0.1,0.8) | (0.1,0.8) | (0.7,0.2) | (0.8,0.2) | (0.7,0.1) | (0.3,0.5) |
| *F*_3_ | (0.7,0.3) | (0.6,0.3) | (0.0,0.0) | (0.3,0.5) | (0.1,0.7) | (0.2,0.7) | (0.7,0.2) | (0.7,0.2) | (0.8,0.1) | (0.4,0.4) |
| *F4* | (0.9,0.1) | (0.1,0.7) | (0.2,0.7) | (0.0,0.0) | (0.2,0.6) | (0.3,0.6) | (0.6,0.2) | (0.1,0.7) | (0.8,0.1) | (0.3,0.5) |
| *F*_5_ | (0.4,0.4) | (0.6,0.2) | (0.5,0.4) | (0.2,0.6) | (0.0,0.0) | (0.3,0.5) | (0.3,0.6) | (0.2,0.7) | (0.2,0.7) | (0.4,0.6) |
| *F*_6_ | (0.5,0.4) | (0.3,0.5) | (0.3,0.6) | (0.2,0.7) | (0.1,0.8) | (0.0,0.0) | (0.2,0.7) | (0.3,0.7) | (0.4,0.5) | (0.4,0.6) |
| *F*_7_ | (0.5,0.3) | (0.4,0.4) | (0.3,0.7) | (0.4,0.4) | (0.7,0.2) | (0.3,0.6) | (0.0,0.0) | (0.3,0.7) | (0.4,0.4) | (0.2,0.7) |
| *F*_8_ | (0.2,0.7) | (0.4,0.4) | (0.2,0.7) | (0.2,0.7) | (0.4,0.6) | (0.1,0.7) | (0.1,0.9) | (0.0,0.0) | (0.0,0.8) | (0.3,0.6) |
| *F*_9_ | (0.4,0.4) | (0.3,0.5) | (0.6,0.3) | (0.2,0.7) | (0.4,0.3) | (0.2,0.7) | (0.2,0.7) | (0.2,0.7) | (0.0,0.0) | (0.2,0.7) |
| *F*_10_ | (0.8,0.1) | (1.0,0.0) | (0.4,0.3) | (0.7,0.2) | (0.7,0.1) | (0.6,0.2) | (0.8,0.2) | (0.8,0.2) | (0.7,0.2) | (0.0,0.0) |

**Table S5. Initial intuitionistic fuzzy direct-relation matrix provided by *E*_5._**

|  | ***F*_1_** | ***F*_2_** | ***F*_3_** | ***F*_4_** | ***F*_5_** | ***F*_6_** | ***F*_7_** | ***F*_8_** | ***F*_9_** | ***F*_10_** |
| --- | --- | --- | --- | --- | --- | --- | --- | --- | --- | --- |
| *F*_1_ | (0.0,0.0) | (0.2,0.7) | (0.1,0.7) | (0.2,0.7) | (0.7,0.1) | (0.7,0.2) | (0.4,0.4) | (0.3,0.6) | (0.8,0.1) | (0.8,0.1) |
| *F*_2_ | (0.7,0.3) | (0.0,0.0) | (0.7,0.1) | (0.1,0.8) | (0.1,0.8) | (0.1,0.7) | (0.6,0.3) | (0.8,0.1) | (0.8,0.1) | (0.4,0.6) |
| *F*_3_ | (0.5,0.4) | (0.7,0.3) | (0.0,0.0) | (0.3,0.6) | (0.1,0.8) | (0.1,0.7) | (0.7,0.2) | (0.5,0.5) | (0.7,0.2) | (0.3,0.6) |
| *F*_4_ | (0.8,0.1) | (0.1,0.8) | (0.3,0.7) | (0.0,0.0) | (0.1,0.8) | (0.1,0.7) | (0.6,0.2) | (0.1,0.7) | (0.7,0.1) | (0.4,0.3) |
| *F*_5_ | (0.5,0.5) | (0.7,0.3) | (0.3,0.3) | (0.2,0.7) | (0.0,0.0) | (0.4,0.4) | (0.3,0.6) | (0.2,0.7) | (0.3,0.5) | (0.5,0.5) |
| *F*_6_ | (0.5,0.4) | (0.2,0.6) | (0.1,0.7) | (0.4,0.6) | (0.2,0.8) | (0.0,0.0) | (0.2,0.7) | (0.2,0.7) | (0.1,0.7) | (0.5,0.5) |
| *F*_7_ | (0.4,0.3) | (0.5,0.4) | (0.2,0.7) | (0.3,0.6) | (0.6,0.3) | (0.2,0.6) | (0.0,0.0) | (0.2,0.7) | (0.4,0.5) | (0.3,0.6) |
| *F*_8_ | (0.2,0.6) | (0.4,0.5) | (0.0,0.8) | (0.4,0.4) | (0.6,0.3) | (0.2,0.7) | (0.2,0.6) | (0.0,0.0) | (0,0.8) | (0.1,0.8) |
| *F*_9_ | (0.5,0.4) | (0.4,0.6) | (0.6,0.3) | (0.2,0.7) | (0.6,0.1) | (0.2,0.7) | (0.2,0.7) | (0.3,0.7) | (0.0,0.0) | (0.3,0.7) |
| *F*_10_ | (0.7,0.2) | (0.7,0.2) | (0.3,0.3) | (0.7,0.1) | (0.8,0.1) | (0.8,0.2) | (0.7,0.2) | (0.9,0.0) | (0.9,0.0) | (0.0,0.0) |

S**Table S6. Initial intuitionistic fuzzy direct-relation matrix provided by *E*_6_.**

|  | ***F*_1_** | ***F*_2_** | ***F*_3_** | ***F*_4_** | ***F*_5_** | ***F*_6_** | ***F*_7_** | ***F*_8_** | ***F*_9_** | ***F*_10_** |
| --- | --- | --- | --- | --- | --- | --- | --- | --- | --- | --- |
| *F*_1_ | (0.0,0.0) | (0.1,0.8) | (0.2,0.7) | (0.3,0.6) | (0.7,0.2) | (0.7,0.2) | (0.5,0.5) | (0.2,0.6) | (0.6,0.3) | (0.8,0.2) |
| *F*_2_ | (0.8,0.1) | (0.0,0.0) | (0.8,0.1) | (0.1,0.8) | (0.1,0.8) | (0.1,0.7) | (0.6,0.2) | (0.5,0.3) | (0.8,0.1) | (0.5,0.5) |
| *F*_3_ | (0.6,0.3) | (0.6,0.3) | (0.0,0.0) | (0.1,0.8) | (0.1,0.7) | (0.2,0.7) | (0.7,0.2) | (0.6,0.4) | (0.9,0.0) | (0.4,0.5) |
| *F*_4_ | (0.8,0.1) | (0.1,0.8) | (0.2,0.6) | (0.0,0.0) | (0.1,0.8) | (0.2,0.6) | (0.7,0.1) | (0.1,0.6) | (0.7,0.2) | (0.4,0.5) |
| *F*_5_ | (0.4,0.4) | (0.5,0.4) | (0.6,0.3) | (0.1,0.8) | (0.0,0.0) | (0.3,0.4) | (0.5,0.4) | (0.1,0.8) | (0.2,0.7) | (0.4,0.4) |
| *F*_6_ | (0.4,0.4) | (0.1,0.8) | (0.2,0.7) | (0.1,0.7) | (0.1,0.7) | (0.0,0.0) | (0.1,0.8) | (0.2,0.8) | (0.2,0.7) | (0.4,0.4) |
| *F*_7_ | (0.5,0.5) | (0.3,0.5) | (0.3,0.6) | (0.5,0.4) | (0.8,0.2) | (0.2,0.7) | (0.0,0.0) | (0.1,0.8) | (0.4,0.5) | (0.1,0.8) |
| *F*_8_ | (0.1,0.8) | (0.3,0.4) | (0.1,0.9) | (0.3,0.6) | (0.4,0.3) | (0.2,0.7) | (0.2,0.7) | (0.0,0.0) | (0.1,0.8) | (0.1,0.8) |
| *F*_9_ | (0.4,0.4) | (0.3,0.6) | (0.6,0.3) | (0.1,0.7) | (0.6,0.3) | (0.2,0.7) | (0.1,0.7) | (0.1,0.7) | (0.0,0.0) | (0.2,0.7) |
| *F*_10_ | (0.8,0.1) | (0.8,0.1) | (0.5,0.4) | (0.8,0.2) | (0.7,0.2) | (0.9,0.0) | (0.8,0.1) | (0.8,0.1) | (0.8,0.1) | (0.0,0.0) |

1. **The values used to build graphs.**

(1) Table S4 is the data of Fig. 2 in the manuscript.

**Table S4. Intuitionistic fuzzy over-relation matrix *T*.**

|  | ***F*_1_** | ***F*_2_** | ***F*_3_** | ***F*_4_** | ***F*_5_** | ***F*_6_** | ***F*_7_** | ***F*_8_** | ***F*_9_** | ***F*_10_** |
| --- | --- | --- | --- | --- | --- | --- | --- | --- | --- | --- |
| *F*_1_ | (0.035,0.021) | (0.049,0.097) | (0.043,0.096) | (0.043,0.099) | (0.103,0.030) | (0.094,0.052) | (0.074,0.061) | (0.044,0.093) | (0.104,0.031) | (0.103,0.044) |
| *F*_2_ | (0.105,0.035) | (0.032,0.024) | (0.098,0.041) | (0.033,0.102) | (0.063,0.077) | (0.035,0.095) | (0.095,0.040) | (0.100,0.041) | (0.109,0.017) | (0.068,0.074) |
| *F*_3_ | (0.092,0.053) | (0.091,0.053) | (0.025,0.032) | (0.046,0.091) | (0.048,0.097) | (0.041,0.095) | (0.097,0.044) | (0.089,0.055) | (0.110,0.020) | (0.068,0.072) |
| *F*_4_ | (0.104,0.038) | (0.032,0.102) | (0.039,0.104) | (0.016,0.040) | (0.037,0.106) | (0.042,0.100) | (0.094,0.040) | (0.031,0.097) | (0.104,0.024) | (0.059,0.078) |
| *F*_5_ | (0.067,0.071) | (0.081,0.061) | (0.067,0.078) | (0.034,0.106) | (0.023,0.033) | (0.057,0.081) | (0.063,0.074) | (0.039,0.108) | (0.052,0.085) | (0.060,0.083) |
| *F*_6_ | (0.063,0.070) | (0.0360.099) | (0.034,0.112) | (0.035,0.115) | (0.033,0.110) | (0.014,0.050) | (0.036,0.106) | (0.033,0.119) | (0.044,0.090) | (0.054,0.092) |
| *F*_7_ | (0.070,0.068) | (0.062,0.074) | (0.041,0.106) | (0.054,0.088) | (0.090,0.056) | (0.039,0.104) | (0.024,0.031) | (0.036,0.105) | (0.068,0.068) | (0.040,0.101) |
| *F*_8_ | (0.031,0.099) | (0.050,0.087) | (0.017,0.134) | (0.033,0.112) | (0.060,0.082) | (0.027,0.116) | (0.038,0.101) | (0.011,0.048) | (0.023,0.103) | (0.025,0.120) |
| *F*_9_ | (0.066,0.072) | (0.055,0.087) | (0.073,0.073) | (0.030,0.110) | (0.069,0.063) | (0.039,0.105) | (0.044,0.093) | (0.041,0.106) | (0.025,0.025) | (0.042,0.105) |
| *F*_10_ | (0.129,0.017) | (0.136,0.005) | (0.079,0.041) | (0.103,0.007) | (0.117,0.019) | (0.109,0.007) | (0.124,0.021) | (0.118,0.006) | (0.131,0.003) | (0.040,0.006) |

(2) Table S5 is the data of Fig. 3 in the manuscript.

**Table S5. Computational results.**

|  | ***c_i_’*** | ***h_i_’*** | ***c_i_*** | ***h_i_*** | ***D_i_*** | ***R_i_*** |
| --- | --- | --- | --- | --- | --- | --- |
| *F*_1_ | (0.514,0.000) | (0.579,0.000) | 0.881 | 0.886 | 1.768 | -0.004 |
| *F*_2_ | (0.537,0.000) | (0.495,0.000) | 0.887 | 0.894 | 1.747 | 0.038 |
| *F*_3_ | (0.522,0.000) | (0.471,0.000) | 0.836 | 0.860 | 1.746 | 0.026 |
| *F*_4_ | (0.440,0.000) | (0.377,0.000) | 0.818 | 0.806 | 1.649 | 0.037 |
| *F*_5_ | (0.429,0.000) | (0.521,0.000) | 0.872 | 0.885 | 1.722 | -0.048 |
| *F*_6_ | (0.323,0.000) | (0.422,0.000) | 0.839 | 0.833 | 1.604 | -0.062 |
| *F*_7_ | (0.418,0.000) | (0.559,0.000) | 0.878 | 0.903 | 1.733 | -0.072 |
| *F*_8_ | (0.275,0.000) | (0.488,0.000) | 0.689 | 0.869 | 1.606 | -0.132 |
| *F*_9_ | (0.393,0.000) | (0.603,0.000) | 0.836 | 0.921 | 1.737 | -0.105 |
| *F*_10_ | (0.685,0.000) | (0.477,0.000) | 0.919 | 0.863 | 1.813 | 0.087 |
